# Chemical Profiles of Serbian *Nepeta cataria* Essential Oils, Structural Characterization of *trans*-*cis*-Nepetalactone, and Preliminary In Vitro Bioactivity

**DOI:** 10.3390/plants15213406

**Published:** 2026-11-02

**Authors:** Jelena S. Lazarević, Miroslava B. Stanković, Milan S. Nešić, Niko S. Radulović, Bojan K. Zlatković, Gordana S. Stojanović

**Affiliations:** 1 Department of Chemistry, Faculty of Medicine, University of Niš, Bulevar Dr Zorana Đinđića 81, 18000 Niš, Serbia; 2 Nuclear Facilities of Serbia, Mike Petrovića Alasa 12–14, Vinča, 11001 Belgrade, Serbia; miroslava.stankovic@nuklearniobjekti.rs; 3 Department of Chemistry, Faculty of Sciences and Mathematics, University of Niš, Višegradska 33, 18000 Niš, Serbia; milan.nesic@pmf.edu.rs (M.S.N.); nikoradulovic@yahoo.com (N.S.R.); gordanastojanovic2411960@gmail.com (G.S.S.); 4 Department of Biology and Ecology, Faculty of Sciences and Mathematics, University of Niš, Višegradska 33, 18000 Niš, Serbia; bojan.zlatkovic@pmf.edu.rs

**Keywords:** *Nepeta cataria* essential oils, NMR spectroscopy, *trans*,*cis*-nepetalactone, lipid peroxidation, micronucleus frequency

## Abstract

Essential oils from three wild populations of *Nepeta cataria* L. (NC_1_–NC_3_) in southern and southeastern Serbia were obtained by hydrodistillation and analyzed by GC-FID and GC-MS. *trans*,*cis*-Nepetalactone (4a*α*,7*α*,7a*β*-nepetalactone; NL) was isolated from NC_1_, and its constitution and relative configuration were established by NMR spectroscopy and iterative full-spin analysis; its absolute configuration was not determined. Its presence across the profiles was confirmed by co-injection with the isolated and characterized pure compound. NL accounted for 91.5%, 12.9%, and 87.9% of the relative GC-FID peak area in NC_1_, NC_2_, and NC_3_, respectively, whereas NC_2_ contained larger proportions of two additional provisionally annotated nepetalactone components, D (41.0%) and E (16.4%), whose configurations remain unresolved. In exploratory in vitro experiments, both the essential oils (NC_1_–NC_3_) and the isolated NL were evaluated using a cell-free lipid peroxidation model (TBA–MDA assay) and a cytokinesis-block micronucleus (CBMN) assay on lymphocytes from four male donors. All tested samples inhibited AAPH-induced lipid peroxidation. Preliminary CBMN screenings revealed a distinct non-monotonic profile: lower concentrations (1–2 µg/mL) reduced baseline micronucleus frequency, while 4 µg/mL caused an increase. These preliminary biological observations do not establish cytoprotection or safety. The findings document marked chemical variation among the analyzed Serbian samples, pointing to a complex, dose-dependent trend that requires confirmation with independent cytotoxicity measurements and a larger donor cohort.

## 1. Introduction

Lipid peroxidation (LP), a process closely related to oxidative stress in living organisms, is attracting intense scientific attention as it initiates ferroptosis, functions as a central mechanism in various age-related diseases, and plays a crucial role in cellular signaling [1]. Mediated by free radicals, LP results in generation of lipid peroxyl radicals and hydroperoxides, which act as the primary driving force during the LP propagation phase. The propagation amplifies an initial radical event into a destructive, self-sustaining chain reaction, that can be blocked/prevented by antioxidants [2]. Once initiated, LP leads to the oxidative deterioration of polyunsaturated lipids and, if excessive, alters the physical properties of cell membranes, contributing to functional loss and modification of proteins and DNA bases, and ultimately to disruption of cell membranes and organelles [3]. Lipid-peroxidation-induced oxidative DNA damage triggers responses critical for genomic stability [4]. When such processes are out of balance, pathophysiological processes may be accelerated, because oxidative DNA damage and resulting mutagenic processes—chromosomal aberrations and mutagenic point mutations—are tightly linked to aging, inflammation, and the development of multiple age-related diseases, such as cancer and neurodegenerative disorders [5].

Micronuclei, forming from chromosome fragments or intact chromosomes that are not included in daughter nuclei during mitosis, are among the most widely studied biomarkers of DNA damage and chromosomal instability in humans [6]. The primary causes of their formation are an absence of functional centromeres in chromosome fragments or entire chromosomes, or defects in one or more proteins of the mitotic system that ultimately result in improper chromosome segregation. Validated assays now quantify micronuclei across diverse cellular matrices; however, the use of peripheral blood lymphocytes has a distinct advantage because of the availability of well-developed techniques to stimulate lymphocytes to divide with mitogens, which enables the expression of micronuclei caused by mitotic errors and structural damage to DNA. The extent of chromosomal DNA damage is successfully measured using the cytokinesis-block micronucleus assay (CBMN). The dependable and reproducible CBMN assay is a standard genotoxicity test for mammalian cells [7], and has been extensively utilized to assess the level of chromosome damage in human populations affected by genotoxic exposure via environmental, occupational, or lifestyle factors and genetic susceptibility to DNA damage, and for evaluating risk of accelerated aging and diseases influenced by genomic instability such as developmental defects and cancer [8]. The CBMN assay has also been used to assess antioxidant and DNA protective effects of various natural compounds and extracts [9,10].

Over the past decades, interest in the therapeutic potential of natural compounds and plant extracts with antioxidant properties has grown. Plant-derived antioxidants have been extensively evaluated for their ability to neutralize harmful radicals without interfering with essential physiological processes, lower the risk of chronic disease, and offer promising therapeutic strategies against oxidative-stress-induced damage [11]. The antioxidant properties of essential oils and extracts from medicinal and aromatic plants have received considerable attention as safe and efficient alternatives to synthetic preservatives, and as promising agents for health-related applications [12].

Numerous diverse pharmacological attributes of *Nepeta* species [13,14,15,16] are linked to their secondary metabolites: essential oil and flavonoids. Many reports on *Nepeta* spp. essential oils revealed that some of them are characterized by the presence of a high content of volatile iridoid monoterpene aglycones (methylcyclopentanoids) called nepetalactones [17,18]. While these metabolites in *Nepeta* plants mediate complex interactions with insects (plant–pollinator and plant–herbivore relationships), phytopathogens (counting antimicrobial activity as well), and neighboring plants (allelopathy and phytotoxicity) [19], they are known for inducing temporary behavioral changes in Felidae-family animals, acting as a feline attractant and mood-altering stimulant that causes euphoria and excitement [20]. Preliminary findings suggest that nepetalactones may hold pharmacological relevance for human medicine [21]. The high degree of compositional variability in *Nepeta* essential oils and especially the variation in the different isomers of nepetalactone influenced by the complexity of eco-geographic factors suggest that nepetalactones could also be used as taxonomic markers [19,22]. Nevertheless, insufficient chemical data for clear chemotaxonomic interpretations make the current understanding of the complex phylogenetic relationships within the genus *Nepeta* still uncertain.

Native to Europe and Asia, and naturalized worldwide due to excellent ecological adaptability [21], a strong-scented perennial *N. cataria* L. is among the most widespread and intensively studied *Nepeta* species. Publications on *N. cataria* essential oils clearly demonstrate that wide chemical polymorphism is characteristic of the species. As reported, *N. cataria* from geographical areas worldwide showed significant variation in essential-oil composition, especially with regard to the accumulation of nepetalactone isomers, which is largely dependent on soil and climatic conditions, seasonal variations, individual stages of plant and plant organ development, insect feeding, the method of raw material processing prior to analysis (e.g., methods of extraction), and the choice of the analytical method used [23].

*Nepeta cataria* also has a history of traditional medicinal use [13], and possess hepatoprotective, antidiabetic, antidepressant, spasmolytic, anti-nociceptive, and anti-inflammatory properties [21]. *Nepeta cataria* oils are rich in nepetalactones and, according to scientific reports, possess antibacterial, antifungal, insecticidal, and antioxidant properties [21]. A literature search on antioxidant activity of *N. cataria* revealed almost exclusive dominance of data on extracts obtained using various polar solvents, whereby the species has been recognized as a rich source of natural antioxidants, mainly due to its high content of phenolic compounds [16,23,24,25]. However, studies examining the activity of *N. cataria* essential oils are less common [26,27,28]. Although the antioxidant properties of *Nepeta* oils are usually attributed to the presence of the oxygenated monoterpenes 1,8-cineole, linalool, or nepetalactone [28,29], surprisingly, none of the published studies have actually examined the activity of any of the isolated stereoisomeric nepetalactones from *N. cataria*, or any other nepetalactone-rich *Nepeta* species. So far, the antioxidant capacity of *N. cataria* essential oils was assessed by simplified chemical assays such as 2,2-diphenyl-1-picrylhydrazyl radical scavenging assay (DPPH), 2,2′-azino-bis(3-ethylbenzothiazoline-6-sulfonic acid) radical cation decolorization assay (ABTS), ferric reducing antioxidant power assay (FRAP), CUPric reducing antioxidant capacity assay (CUPRAC), oxygen radical absorbance capacity assay (ORAC), and beta-carotene/linoleic acid assays, often offering convenience and a rapid antioxidant potency screening [26,27,29,30,31].

Considering the varied biological functions of *Nepeta* essential oils and the extensive number of encouraging studies on the distinct biological activities of iridoid-rich species assessed to date, along with the lack of literature data regarding the antioxidant properties of oils/nepetalactone(s), this study aimed to analyze and compare the chemical profiles of wild *N. cataria* populations from southern and southeastern Serbia. Furthermore, the study aimed to isolate pure nepetalactone(s) which, after structural characterization, together with the oil samples, were subjected to preliminary bioactivity screenings. This study provides a detailed approach combining the GC-FID and GC-MS characterization of three *N. cataria* oil samples (NC_1_–NC_3_), the isolation and NMR validation of *trans*,*cis*-nepetalactone (4aα,7α,7aβ-nepetalactone; NL), and an evaluation of their effects utilizing a cell-free lipid peroxidation model (TBA–MDA assay) and a human lymphocyte micronucleus frequency protocol (CBMN assay). This comprehensive investigation was exploratory in nature, designed to provide a novel, integrative chemical and preliminary biological screening rather than establishing stable population chemotypes or defining definitive molecular mechanisms.

## 2. Results and Discussion

### 2.1. Nepeta cataria Essential Oil Characterization and Spectral Identification of the Isolated Nepetalactone NL

The essential oils were isolated by hydrodistillation from the aerial parts of three geographically distinct wild populations of *N. cataria* L. originating from Serbia (Table 1). The plant material gathered during the flowering phase was distilled fresh to obtain the volatile oils, which, prior to compositional analyses, were subjected to the standard preparation procedure as described in Section 4. All NC_1_–NC_3_ oils were pale yellow and fragrant.

Chromatographic fractionation of NC_1_, followed by preparative TLC, afforded 150 mg of NL, whose constitution and relative configuration were established by NMR spectroscopy and full-spin analysis. NC_2_ and NC_3_ were also subjected to chromatographic fractionation using procedures analogous to those applied to NC_1_, with the aim of isolating additional nepetalactone diastereomers. No additional diastereomer was successfully isolated under the conditions used. The assignment of NL in each oil was verified by comparison with the NMR-characterized isolate from NC_1_, including co-injection. The structural characterization of NL is discussed below, followed by the composition of the three oils.

**Figure 1:**
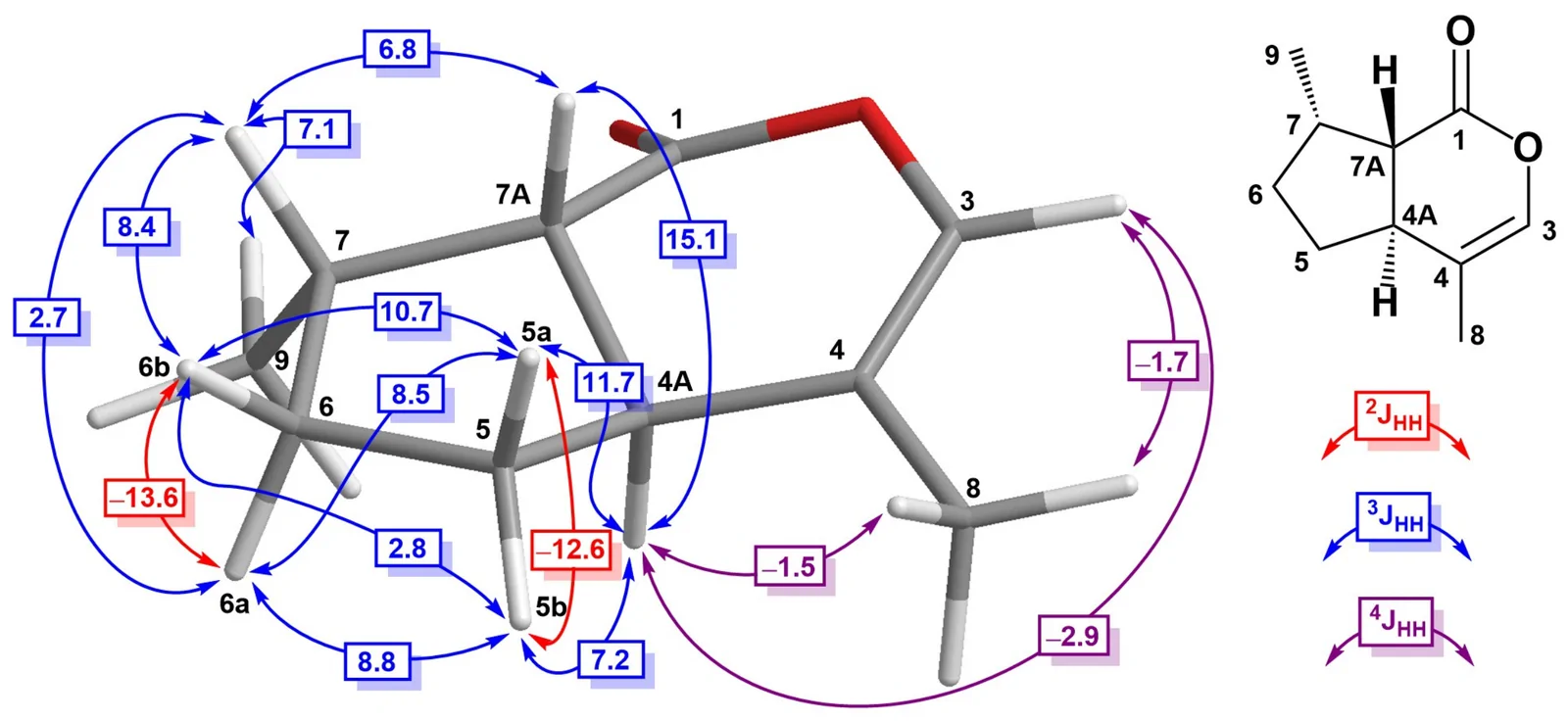
Selected observed ^1^H–^1^H coupling constants represented on 3D model of the isolated **NC_1_** compound, 4a*α*,7*α*,7a*β*-nepetalactone (IUPAC: *rel*-(4a*S*,7*S*,7a*S*)-4,7-dimethyl-5,6,7,7a-tetrahydrocyclopenta[c]pyran-1(4a*H*)-one; *trans*,cis-nepetalactone; NL). Absolute configuration and enantiomeric composition were not determined.

The combined analysis of the ^1^H–^1^H and ^1^H–^13^C coupling constants, together with the NOESY correlations (Table 2 and Table S1, Supplementary Material), supports a rigid and conformationally constrained structure of compound NL—4a*α*,7*α*,7a*β*-nepetalactone (*trans*-*cis* nepetalactone). The exceptionally large vicinal coupling constant between H-4A and H-7A (^3^*J*_4A,7A_ = 15.1 Hz, Figure 1) is consistent with an almost antiperiplanar arrangement of these protons within the fused ring system. A similar conclusion can be drawn from the large ^3^*J*_4A,5a_ value of 11.7 Hz, which indicates a fixed anti relationship between H-4A and H-5a. The remaining vicinal ^1^H–^1^H coupling constants follow the geometry expected from the 3D model: large or moderately large values were observed for H-5a/H-6b, H-6b/H-7, and H-7/H-7A (10.7, 8.4, and 6.8 Hz, respectively), whereas, for example, much smaller couplings were found for H-6a/H-7—2.7 Hz. This coupling pattern defines the relative orientation of H-7 and, consequently, the opposite orientation of the C-9 methyl group. The stereochemical assignment is further supported by the NOESY data, which define two spatially related proton sets, H-4A/H-5b/H-6a/H-9 and H-5a/H-6b/H-7/H-7A, located on opposite faces of the molecule.

The rigid geometry and restricted conformational freedom of the molecule are further supported by the allylic coupling constant of −2.9 Hz between H-3 and H-4A, which is consistent with an approximately orthogonal arrangement of the interacting bonds relative to the C3=C4 π-system. The remaining four-bond couplings transmitted through the π-system, ^4^*J*_3,8_ and ^4^*J*_4A,8_, had the expected smaller magnitudes of −1.7 and −1.5 Hz, respectively.

In addition to the conventional ^13^C NMR spectrum, the coupled ^13^C spectrum and its partial-spin simulation (Figure S7, Supplementary Material) enabled the identification of several one-bond and long-range ^1^H–^13^C coupling constants. The heteronuclear couplings provide additional checks on connectivity and local geometry; they are treated as supporting evidence rather than an independent determination of configuration. The ^3^*J*_H-7A,C-9_ value of 7.8 Hz (Figure S5, Supplementary Material) is relatively large and is consistent with the spatial arrangement of H-7A and the C-9 methyl carbon, whereas the smaller ^3^*J*_H-6b,C-9_ value of 3.0 Hz reflects a less favorable coupling geometry. The partially overlapping 5.6 Hz contribution assigned to ^3^*J*_H-6a,C-9_ and ^2^*J*_H-7,C-9_ is also compatible with the local geometry around C-7. Overall, the resolved two- and three-bond ^1^H–^13^C coupling constants are in good agreement with the proposed structure and with the dihedral relationships estimated from the 3D model. The relatively small ^3^*J*_H-3,C-8_ value of 3.6 Hz, observed across the C3=C4–C8 fragment, may be rationalized by the combined effects of the local alkene geometry and the adjacent oxygen atom. Conversely, the high one-bond coupling constant ^1^*J*_H-3,C-3_ = 195.5 Hz is consistent with the electron-withdrawing influence of oxygen on the vinylic C-3 carbon.

GC-FID and GC-MS analyses enabled the detection of 147 components, representing 98.6–99.7% of the overall oils’ content. The NC_1_–NC_3_ essential-oil profiles are presented in Table 3. The constitution and relative configuration of NL isolated from NC_1_ were established by NMR spectroscopy and full-spin analysis. Its presence in all three oils was confirmed by chromatographic and mass-spectral comparison and co-injection with the NMR-characterized isolated compound. NL accounted for 91.5%, 12.9%, and 87.9% of the relative GC-FID peak area in NC_1_, NC_2_, and NC_3_, respectively. Thus, NC_1_ and NC_3_ were dominated by the same relative nepetalactone isomer, whereas NC_2_ contained larger proportions of components D (41.0%) and E (16.4%).

Attempts to isolate additional nepetalactone diastereomers from NC_2_ and NC_3_ were unsuccessful. Consequently, the configurations of components A, D, and E remain unresolved, and they have been provisionally annotated as nepetalactone-related constituents, without assignment of specific configurations. The most abundant component of NC_2_ was D (41.0%), followed by E (16.4%) and NL (12.9%). Component A contributed 0.5% of NC_2_ and was not detected in NC_1_ or NC_3_. Components D and E were also present in NC_1_ at 0.4% and 1.0%, respectively, and in NC_3_ at 0.8% and 3.3%. Their chromatographic and mass-spectral data supported provisional annotation as nepetalactone-related constituents, but did not establish their specific configurations. The letters A, D, and E serve only as peak identifiers.

The sesquiterpene (*E*)-caryophyllene accounted for 2.3%, 8.3%, and 4.0% in NC_1_, NC_2_, and NC_3_, respectively. The principal difference among these samples was therefore the relative distribution of the major nepetalactone-related constituents. NC_1_ and NC_3_ had closely similar dominant-component profiles, while NC_2_ contained larger contributions from D and E and a smaller contribution from NL. These observations apply to the three pooled samples analyzed. A single sample per locality does not establish stable population chemotypes or distinguish genetic, environmental, and individual sources of variation. Repeated sampling of individual plants and populations would be required to examine the persistence and origin of these compositional differences.

The terpenoid fraction represented 93.9–98.8% of the total oil, unevenly distributed between mono-, sesqui-, and diterpenoids (75.8–94.4%, 3.4–14.8%, and tr–0.9%, respectively, Table 3). In all three NC_1_–NC_3_ samples, the monoterpenoid fraction consisted almost exclusively of cyclic iridoids (or iridanes), a non-canonical monoterpenoid class with cyclopenta[c]pyran skeleton (70.9–93.0%). The low concentrations of other terpenoids detected in the oils were likely due to the high content of iridanes, as previously observed by Handjieva et al. [32]. *Nepeta cataria* essential oils exhibited complex iridoid profiles, with six to nine distinct iridoids detected across the analyzed samples (Table 3, MI class members). The iridoid composition of NC_2_ was more complex than that of NC_1_ and NC_3_, which were both characterized by a high abundance of the frequently reported *N. cataria* nepetalactones [32,33].

In an informal olfactory assessment, NC_1_ and NC_3_ were described as sweet and pleasant, whereas NC_2_ had a more pungent odor. These observations were not obtained using a standardized sensory protocol and cannot be attributed to individual constituents or specific stereoisomers.

**Table 3:** Chemical composition of *Nepeta cataria* essential oils (NC_1_–NC_3_) determined by GC-FID (quantitative) and GC-MS (qualitative) analysis.

RI ^1^	Compound ^2^	NC_1_	NC_2_	NC_3_	Class ^3^	ID ^4^
762	Pentan-1-ol	-	tr	-	O	a,b
765	(*Z*)-2-Penten-1-ol	tr	tr	tr	O	a,b
778	Methyl 2-methylbutanoate	-	tr	-	O	a,b,c
800	(*Z*)-3-Hexenal ^5^	-	tr	-	O	a,b
801	Hexanal	-	tr	-	O	a,b,c
822	(*Z*,*E*)-1,3,6-Octatriene	-	tr	-	O	a,b
833	Furfural ^5^	tr	tr	tr	O	a,b,c
846	(*E*)-2-Hexenal ^5^	0.1	0.1	-	O	a,b
852	(*Z*)-3-Hexen-1-ol	-	1.6	0.4	O	a,b
860	(*Z*)-2-Hexen-1-ol	tr	0.2	tr	O	a,b
862	Hexan-1-ol	-	tr	-	O	a,b
877	(*E*,*E*)-1,3,6-Octatriene	-	tr	-	O	a,b
897	Styrene ^6^	tr	0.4	0.1	O	a,b,c
900	Nonane	0.1	-	-	O	a,b,c
902	Heptanal	-	tr	-	O	a,b
911	(*E*,*E*)-2,4-Hexadienal ^5^	-	tr	-	O	a,b
928	*α*-Thujene	tr	tr	tr	T/M	a,b,c
936	*α*-Pinene	0.1	0.2	tr	T/M	a,b,c
952	Camphene	-	tr	-	T/M	a,b,c
955	(*E*)-2-Heptenal ^5^	-	tr	-	O	a,b
963	Benzaldehyde	tr	tr	-	O	a,b,c
966	Heptan-1-ol	-	tr	-	O	a,b
976	Sabinene	0.2	0.9	0.2	T/M	a,b,c
977	1-Octene-3-ol	tr	tr	tr	O	a,b
982	*β*-Pinene	0.4	1.9	0.4	T/M	a,b,c
985	3-Octanone	tr	tr	-	O	a,b
990	Myrcene	tr	0.1	tr	T/M	a,b,c
991	2-Pentyl furan ^5^	tr	tr	-	O	a,b
995	3-Octanol	-	tr	-	O	a,b
997	Dehydro-1,8-cineole	-	tr	-	T/M	a,b
999	(*Z*,*E*)-2,4-Heptadienal ^5^	tr	tr	-	O	a,b
1003	Octanal	tr	0.1	tr	O	a,b,c
1005	(*E*)-3-Hexenyl acetate	-	0.1	-	O	a,b
1012	(*E*,*E*)-2,4-Heptadienal ^5^	tr	tr	-	O	a,b
1019	*α*-Terpinene	tr	tr	tr	T/M	a,b,c
1026	*p*-Cymene	tr	tr	tr	T/M	a,b,c
1030	Limonene	-	tr	tr	T/M	a,b,c
1031	*β*-Phellandrene	0.1	0.1	tr	T/M	a,b,c
1034	1,8-Cineole	tr	-	-	T/M	a,b,c
1035	(*Z*)-*β*-Ocimene	0.1	0.2	tr	T/M	a,b
1042	3-Ethyl-2-methyl-1,3-heptadiene	-	0.2	tr	O	a,b
1045	2-Phenylacetaldehyde	-	tr	-	O	a,b,c
1046	(*E*)-*β*-Ocimene	0.4	0.7	0.1	T/M	a,b
1057	(*E*)-2-Octenal ^5^	tr	tr	-	O	a,b
1059	*γ*-Terpinene	tr	tr	tr	T/M	a,b
1066	(*E*)-2-Octen-1-ol	tr	tr	-	O	a,b
1068	1-Octanol	tr	0.5	tr	O	a,b,c
1069	*cis*-Sabinene hydrate	tr	tr	tr	T/M	a,b
1074	*cis*-Linalool oxide (furanoid)	tr	tr	tr	T/M	a,b,c
1091	Terpinolene	tr	tr	-	T/M	a,b
1099	Methyl benzoate	tr	tr	-	O	a,b,c
1101	Linalool	tr	tr	tr	T/M	a,b,c
1103	*trans*-Sabinene hydrate	tr	0.1	-	T/M	a,b
1104	Nonanal	tr	0.1	tr	O	a,b,c
1111	*α*-Thujone	-	-	tr	T/M	a,b,c
1125	Dehydrosabina ketone	tr	tr	-	T/M	a,b
1129	*α*-Campholenal	tr	tr	-	T/M	a,b
1142	Nopinone	tr	0.1	tr	T/M	a,b
1143	*trans*-Sabinol	tr	tr	-	T/M	a,b,c
1146	*cis*-Pinene hydrate	-	tr	-	T/M	a,b
1151	Camphor	tr	tr	tr	T/M	a,b,c
1153	Citronellal	tr	tr	-	T/M	a,b
1159	(*E*)-2-Nonenal ^5^	-	tr	-	O	a,b
1162	Sabina ketone	-	tr	tr	T/M	a,b
1167	Lavandulol	0.1	0.2	0.5	T/M	a,b
1181	Terpinen-4-ol	tr	0.1	tr	T/M	a,b,c
1194	*α*-Terpineol	tr	tr	tr	T/M	a,b,c
1199	Methyl salicylate	0.1	0.1	0.1	O	a,b,c
1201	Myrtenal	tr	0.1	-	T/M	a,b,c
1204	Decanal	0.3	0.4	tr	O	a,b,c
1211	Octyl acetate	-	tr	-	O	a,b
1225	*β*-Cyclocitral	0.5	1.8	0.5	T	a,b
1228	(*Z*)-3-Hexenyl 2-methylbutanoate	tr	tr	-	O	a,b
1235	(*Z*)-3-Hexenyl 3-methylbutanoate	tr	tr	tr	O	a,b
1243	Neral	tr	tr	tr	T/M	a,b,c
1246	Hexyl 3-methylbutanoate	0.1	tr	-	O	a,b,c
1255	Geraniol	tr	tr	-	T/M	a,b,c
1262	(*E*)-2-Decenal	-	tr	-	O	a,b
1271	1-Decanol	-	0.1	tr	O	a,b
1272	Geranial	tr	-	-	T/M	a,b,c
1300	Tridecane	tr	tr	tr	O	a,b,c
1318	(*E*,*E*)-2,4-Decadienal ^5^	-	tr	-	O	a,b
1319	Nepetalactol isomer A ^7,^*	-	-	tr	T/MI	a,b
1323	Vinylguajacol	tr	tr	-	O	a,b
1325	(*E*)-3-Hexenyl tiglate	-	tr	-	O	a,b
1341	Nepetalactol isomer B ^8,^*	-	-	tr	T/MI	a,b
1358	Nepetalactone isomer A ^9,^*	-	0.5	-	T/MI	a,b
1384	NL (*trans*,*cis*-Nepetalactone) ^10,^**	91.5	12.9	87.9	T/MI	a,b,c
1389	(*Z*)-3-Hexenyl (*Z*)-3-hexenoate	0.4	tr	0.3	O	a,b
1390	*β*-Bourbonene	-	0.6	tr	T/S	a,b
1392	(*E*)-*β*-Damascenone	0.1	0.1	-	T	a,b
1394	*β*-Cubebene	-	-	tr	T/S	a,b
1399	Nepetalactone isomer D ^11,^*	0.4	41.0	0.8	T/MI	a,b
1404	Nepetalactone isomer E ^12,^*	1.0	16.4	3.3	T/MI	a,b
1412	Isocaryophyllene	tr	-	tr	T/S	a,b
1419	*α*-*cis*-Bergamotene	tr	tr	tr	T/S	a,b
1422	(*E*)-*β*-Damascone	tr	0.1	-	T	a,b
1424	Dihydronepetalactone isomer A ^13,^*	-	tr	tr	T/MI	a,b
1426	(*E*)-Caryophyllene	2.3	8.3	4.0	T/S	a,b,c
1435	*β*-Copaene	-	tr	-	T/S	a,b
1445	Iridomyrmecin-type compound ^14^	0.1	0.1	0.1	T/MI	a,b
1454	Geranyl acetone	tr	tr	-	T	a,b
1458	(*E*)-*β*-Farnesene	0.2	0.3	tr	T/S	a,b
1460	*α*-Humulene	0.2	1.0	0.4	T/S	a,b
1461	2-Methyltetradecane	-	tr	-	O	a,b
1467	Dihydronepetalactone isomer B ^15,^*	tr	tr	tr	T/MI	a,b
1472	*γ*-Dodecalactone	-	tr	-	O	a,b
1481	putative iridoid ^16^	-	0.1	-	T/MI	b
1487	Germacrene D	tr	0.2	tr	T/S	a,b
1490	(*E*)-*β*-Ionone	tr	0.3	tr	T	a,b,c
1501	Benzyl tiglate	-	tr	-	O	a,b
1509	(*E*,*E*)-*α*-Farnesene	tr	tr	tr	T/S	a,b
1512	Germacrene A	-	tr	-	T/S	a,b
1523	*endo*-1-Bourbonanol	tr	tr	-	T/S	a,b
1533	putative iridoid ^17^	tr	0.1	0.1	T/MI	b
1545	*trans*-*α*-Bisabolene	-	0.1	-	T/S	a,b
1548	*α*-Calacorene	-	tr	-	T/S	a,b
1559	Isocaryophyllene oxide	tr	0.1	tr	T/S	a,b
1574	(*Z*)-3-Hexenyl benzoate	-	0.3	tr	O	a,b
1580	Hexyl benzoate	-	0.1	-	O	a,b,c
1590	Caryophyllene oxide	0.7	3.2	0.5	T/S	a,b,c
1607	Humulene epoxide I	-	0.1	-	T/S	a,b
1612	Tetradecanal	-	tr	-	O	a,b
1616	Humulene epoxide II	tr	0.2	tr	T/S	a,b
1639	Caryophylladienol isomer I	tr	0.1	tr	T/S	a,b
1644	Caryophylladienol isomer II	-	0.3	-	T/S	a,b
1650	*α*-Muurolol (synonym torreyol)	-	0.1	-	T/S	a,b
1672	(*Z*)-3-Hexenyl salicylate	-	tr	-	O	a,b
1676	14-Hydroxycaryophyllene	tr	0.2	-	T/S	a,b
1686	*α*-Bisabolol	-	tr	-	T/S	a,b,c
1713	Pentadecanal	-	tr	-	O	a,b
1723	14-Hydroxy-α-humulene	-	tr	-	T/S	a,b
1768	Benzyl benzoate	tr	0.1	tr	O	a,b,c
1773	14-Hydroxy-*α*-muurolene	-	tr	-	T/S	a,b
1844	Hexahydrofarnesyl acetone	tr	0.1	-	T	a,b
1893	(*Z*,*Z*,*Z*)-8,11,14-Heptadecatrienal	-	tr	-	O	a,b
1960	Hexadecanoic acid	-	tr	-	O	a,b
2081	Abietatriene	-	tr	tr	T/D	a,b
2100	Heneicosane	-	tr	-	O	a,b,c
2117	(*E*)-Phytol	tr	0.9	-	T/D	a,b
2127	Abietadiene	-	tr	-	T/D	a,b
2200	Docosane	-	tr	-	O	a,b,c
2297	Tricosane	tr	tr	-	O	a,b,c
2400	Tetracosane	tr	0.1	tr	O	a,b,c
2500	Pentacosane	tr	0.1	tr	O	a,b,c
2600	Hexacosane	tr	0.1	tr	O	a,b,c
2800	Octacosane	-	tr	-	O	a,b,c
Total		99.5	98.6	99.7		
Essential-oil yield (%, *w*/*w*, fresh weight)		0.12	0.05	0.12		
Grouped components						
Terpenoids and norisoprenoids (terpenoid derivatives) (T)		98.4	93.9	98.8		
Monoterpenoids (M)		94.4	75.8	93.4		
	Monoterpene hydrocarbons	1.3	4.1	0.7		
	Oxygenated monoterpenes ^18^	0.1	0.6	0.5		
	Iridanes (MI)	93.0	70.9	92.1		
Sesquiterpenoids (S)		3.4	14.8	4.9		
	Sesquiterpene hydrocarbons	2.7	10.5	4.4		
	Oxygenated sesquiterpenes	0.7	4.3	0.5		
Diterpenoids (D)		tr	0.9	tr		
Others (O)		1.1	4.7	0.9		

^1^ Constituents are listed in order of elution from a DB-5MS column with retention indices (RI) determined experimentally by co-injection of a homologous series of C_7_–C_40_ *n*-alkanes. ^2^ Values are relative GC-FID peak-area percentages representing the mean of three individual analyses, calculated without compound-specific response factors (i.e., they do not represent calibrated mass fractions); tr-trace (<0.05%); -, not detected in the analysis of oil sample. ^3^ Identified compounds classified into groups according to respective structural types. ^4^ Methods of identification: a, RI matching with literature data; b, mass spectrum matching with data from the literature; c, co-injection with an authentic standard. ^5^ An artifact of hydrodistillation and/or oxidation. ^6^ Probably a contaminant of the isolation procedure. ^7^ MS, 70 eV, 230 °C, *m*/*z* (rel. int.): 168 (89), 97 (94), 84 (76), 83 (77), 81 (100), 71 (82), 67 (73), 41 (97), 39 (76) [34]; ^8^ MS (*m*/*z*): 168 (100), 135 (92), 108 (86), 97 (92), 79 (87), 71 (81), 67 (85), 58 (79), 55 (89), 43 (84) [34]; ^9^ MS (*m*/*z*): 166 (85), 123 (100), 109 (65), 95 (88), 81 (97), 80 (46), 69 (87), 67 (55), 41 (56), 39 (47) [35]; ^10^ MS (*m*/*z*): 166 (98), 123 (100), 109 (62), 95 (89), 81 (99), 80 (44), 69 (75), 67 (65), 41 (49), 39 (47) [35]; ^11^ MS (*m*/*z*): 166 (74), 123 (100), 109 (56), 95 (83), 81 (93), 80 (45), 69 (69), 67 (48), 41 (50), 39 (42) [35]; ^12^ MS (*m*/*z*): 166 (91), 123 (100), 109 (57), 95 (79), 81 (92), 80 (40), 69 (80), 67 (60), 41 (51), 39 (45) [35]; ^13^ MS (*m*/*z*): 168 (6), 154 (6), 153 (54), 139 (4), 127 (10), 126 (18), 125 (11), 114 (12), 113 (100), 100 (6) [36,37,38,39]; ^14^ MS (*m*/*z*): 109 (26), 95 (48), 82 (28), 81 (100), 69 (20), 68 (47), 67 (81), 55 (23), 41 (29), 39 (17) [36,37]; ^15^ MS (*m*/*z*): 153 (64), 113 (58), 109 (20), 95 (30), 82 (39), 81 (100), 68 (42), 55 (37), 53 (21), 41 (35) [36,37,38]; ^16^ MS (*m*/*z*): 109 (100), 96 (74), 87 (62), 81 (98), 67 (47), 69 (35), 55 (40), 43 (36), 41 (45), 39 (31) [37,38,39,40,41]; ^17^ MS (*m*/*z*): 109 (49), 96 (99), 87 (91), 81 (100), 82 (38), 69 (43), 67 (61), 58 (38), 55 (41), 41 (50) [37,38,39,40,41]. ^18^ Standard oxygenated non-iridoid monoterpenes; * the letters A, B, D, and E serve as peak identifiers for additional, provisionally annotated nepetalactone-related constituents whose configurations were not determined; ** denotes nepetalactone NL isolated from NC_1_, whose constitution and relative configuration were established by NMR spectroscopy. Its assignment in NC_1_, NC_2_, and NC_3_ was verified by chromatographic and mass-spectral comparison and by co-injection of each oil with the isolated reference compound.

### 2.2. In Vitro Biological Studies Assaying Nepeta cataria Essential Oils and Nepetalactone NL

#### 2.2.1. TBA–MDA Lipid Peroxidation Assay

Following the methodology as described in Brizzolari et al. [42], the LP inhibition effect of three *N. cataria* oil samples (NC_1_–NC_3_) and of pure NL isolated from NC_1_ was measured, using the method based on TBA–MDA assay, selecting caffeic acid and Trolox as positive controls. To ensure reproducibility, all samples were evaluated across three independent experiments (*N* = 3). For each independent experiment, a minimum of four concentrations were tested in triplicate (three parallel measurements (*n* = 3) per concentration), totaling at least 36 data points per sample (three independent experimental runs × 4 concentrations × 3 technical replicates). The same experiments were performed using known antioxidants caffeic acid and Trolox as standards. Ultimately, the NC_1_–NC_3_ samples were evaluated at final concentrations ranging from 1000 to 32.5 mg/L, whereas pure NL was assayed across a lower concentration range from 160 to 2.5 mg/L. For comparison, the reference standards were tested in the final reaction mixture at lower concentration ranges, optimized for their respective activities: 9.0 to 0.6 mg/L for caffeic acid and 18.7 to 1.2 mg/L for Trolox. The tested oils reduced the TBA-reactive signal in the AAPH-initiated phospholipid oxidation model. IC_50_ values for the tested samples (NC_1_–NC_3_), pure isolated compound (NL), and standards (caffeic acid and Trolox), along with the standard errors (SE) of the 4-PL estimate and 95% confidence intervals (95% CI), are summarized in Table 4. The fitted oil IC_50_ point estimates were 102.5, 160.3, and 272.1 mg/L for NC_1_, NC_3_, and NC_2_, respectively. Their large standard errors indicate substantial uncertainty, and the numerical ordering does not establish differences in potency among the oils. Isolated NL yielded a fitted IC_50_ of 23.9 mg/L (143.8 µM; 95% CI, 22.7–25.2 mg/L), compared with 3.0 mg/L for caffeic acid and 6.3 mg/L for Trolox under the same assay conditions. Isolated NL inhibited AAPH-induced LP; however, this inhibition was weaker than that obtained with the well-known antioxidants used as standards (Table 4).

NL was the major assigned constituent of both NC_1_ and NC_3_, accounting for 91.5% and 87.9% of their GC-FID peak areas, respectively, whereas its contribution to NC_2_ was 12.9%. Isolated NL had a lower fitted IC_50_ than the whole oils on a total sample-mass basis. However, the large standard errors of the oil estimates limit comparisons of potency. These results do not quantify the contribution of NL to the activity of each oil, because relative GC-FID peak areas do not define calibrated constituent doses, and the effects of other constituents and sample behavior in the assay were not separated. The origin of the differences between the oils and isolated NL remains unresolved; matrix effects, synergy, and antagonism were not established.

The reduced TBA-reactive signal is consistent with inhibition of AAPH-initiated lipid oxidation in the cell-free phospholipid model. This endpoint does not identify the reaction pathway responsible for the response or demonstrate direct peroxyl-radical scavenging by NL. Cellular antioxidant signaling cannot account for an effect measured in this cell-free system. The results therefore support a description of the measured lipid-oxidation endpoint, while the underlying mechanism and the contributions of individual oil constituents remain to be established.

#### 2.2.2. In Vitro Cytokinesis-Block Micronucleus (CBMN) Assay

Development of the CBMN methodology by Fenech and Morley [43] by addition of the actin inhibitor cytochalasin-B during the targeted in vitro mitosis allowed the identification of once-divided nuclei as binucleates and provided an efficient approach to study the mechanism leading to the induction of micronuclei. In the CBMN assay, cells that have completed one nuclear division are blocked from performing cytokinesis using cytochalasin-B and are consequently readily identified by their binucleated appearance. Cytochalasin-B is an inhibitor of actin polymerization required for the formation of the microfilament ring that constricts the cytoplasm between the daughter nuclei during cytokinesis [44]. The use of cytochalasin-B enables the accumulation of virtually all dividing cells at the binucleate stage in dividing cell populations regardless of their degree of synchrony and the proportion of dividing cells [45]. Micronuclei are then scored in binucleated cells only, which enables reliable comparison of chromosome damage between populations that may differ in their cell division kinetics.

The concentration-dependent effects of *N. cataria* essential oils (NC_1_–NC_3_) and isolated NL on micronucleus frequency were evaluated in cultured human lymphocytes utilizing the cytokinesis-block micronucleus (CBMN) assay of Fenech and Morley [43,46], incorporating modifications as described in Stanković et al. [9]. The samples were tested across three different concentrations (1.0, 2.0, and 4 µg/mL), and the frequencies along with the distribution of micronuclei in human lymphocytes were documented [43]. Data per individual donors were averaged prior to statistical comparison. Results are expressed as the mean ± standard deviation (SD) calculated from four biological replicates (donors) to reflect inter-individual response dispersion (inter-donor variability, *n* = 4). Table 5 details these experimental findings, including comparative analyses against the reference cytoprotective adjuvant amifostine (WR-2721), the positive genotoxic control mitomycin C (MMC, a micronucleus inducer), and an untreated negative control (blank).

Three out of four tested samples—NC_1_, NC_2_**,** and NL—at a concentration of 1.0 μg/mL reached peak activity (reduction in micronucleus frequency) in comparison to the control cell culture, exhibiting reduction in micronucleus frequency by 30.7%, 27.2%, and 21.9% (*p* < 0.01), respectively. The effect expressed by NC_1_ and NC_3_ at a concentration of 2.0 μg/mL was also a reduction in the micronucleus frequency but to a lesser extent, by 23.2% and 28.3% (*p* < 0.01) relative to the control cultures (Table 5). Treatment with NC_2_ at a concentration of 2.0 µg/mL exhibited effects comparable to those of amifostine. In contrast, cell cultures treated with NC_1_–NC_3_ and NL at 4 µg/mL exhibited an increased incidence of micronucleus frequency relative to control, indicating potential concentration-dependent effects on micronucleus frequency or other adverse effect.

In this exploratory four-donor experiment, lower mean micronucleus frequencies were observed in most conditions tested at 1–2 µg/mL, whereas the mean values at 4 µg/mL were higher than the untreated-control mean for all four preparations (Table 5). Isolated NL at 2 µg/mL gave a value close to that of the untreated control. The limited concentration range and donor number do not establish the mechanism or generality of this response pattern.

CBPI was used to assess proliferative progression, but this endpoint alone cannot exclude cell death or selective loss of damaged cells. A reduction in spontaneous micronucleus frequency cannot therefore be interpreted as demonstrated cytoprotection or evidence of absence of genotoxic potential. The experimental design did not include combined treatment with a test preparation and a genotoxic challenge. Moreover, the cell-free lipid-oxidation results do not establish an intracellular antioxidant mechanism for the lymphocyte findings.

NC_1_ and NC_3_ shared the same assigned major nepetalactone, but comparisons at equal total oil concentration do not provide equal constituent exposures. The present data do not identify which constituents account for the cellular responses. Further studies with verified constituent concentrations, complementary cytotoxicity measurements, a broader concentration range, and replicated donor cultures are needed to examine these observations.

## 3. Materials and Methods

### 3.1. Chemicals

All reagents used herein (magnesium sulphate, sodium hydroxide, sulfuric acid, sodium chloride, thiobarbituric acid (TBA), 2,2′-azobis(2-methylpropionamidine)dihydrochloride (AAPH), trichloroacetic acid (TCA), butylated hydroxytoluene (BHT), mitomycin C (MMC), amifostine WR-2721, cytochalasin B, Giemsa, Gibco^TM^ PB-MAX^TM^ Karyotyping Medium, NMR (tetramethylsilane (TMS))), as well as antioxidant standards (caffeic acid and Trolox) and HPLC grade solvents (diethyl ether, deuterated chloroform (chloroform-d, CDCl_3_), ethyl acetate (EtOAc), *n*-hexane, and methanol) were obtained from commercial sources (Sigma-Aldrich, St. Louis, MO, USA; Merck, Darmstadt, Germany; Carl Roth, Karlsruhe, Germany; Bristol-Myers Squibb, Princeton, NJ, USA; Marligen-Biosciences, Ijamsville, MD, USA; Invitrogen-Gibco-BRL, Vienna, Austria; Thermo Fisher Scientific, Waltham, MA, USA) and used as received, without further purification. Phospholipids (Phospholipon^®^ 90, PL90) were obtained courtesy of Phospholipid GMBH, Cologne, Germany. According to the manufacturer’s specification, the PL90 mixture is composed of phosphatidylcholine 98% and lyso-phosphatidylcholine 2.1%, with the fatty acid composition: palmitic acid 12 ± 2%, stearic acid 3 ± 1%, oleic acid 10 ± 3%, linoleic acid 66 ± 5% and linolenic acid 5 ± 2%, and a maximum peroxide value data amount of 1.3.

Analytical thin-layer chromatography (TLC) was carried out on ALUGRAM^®^ Xtra SIL G/UV_254_ precoated TLC aluminum plates (Macherey-Nagel, Düren, Germany). For preparative chromatography, Silica Gel 60 (particle size 20–40 μm, for preparative column chromatography) and Silica Gel PF_256_ (particle size 5–40 μm, for TLC preparative chromatography) were used as adsorbents.

### 3.2. Plant Material

Three wild populations of *N. cataria* from southern and southeastern Serbia were sampled for an exploratory comparison of their essential-oil profiles. The sampling localities were Mt. Rudina, Izvor, Bosilegrad, southeastern Serbia (sample NC_1_); the vicinity of Niš, southern Serbia (sample NC_2_); and Buštranje, Pčinja District, southern Serbia (sample NC_3_). Above-ground parts were collected in July 2020 at the full-flowering stage at each locality, with the aim of reducing differences associated with developmental stage. Material from multiple individual plants at each locality was combined into a single pooled sample.

Voucher specimens were deposited in the Herbarium of the Faculty of Sciences and Mathematics, Department of Biology and Ecology, University of Niš (HMN), under accession numbers HMN18955–HMN18957. For NC_1_, 1200 g of fresh plant material was subjected to hydrodistillation, while approximately 600 g was used for each of NC_2_ and NC_3_. With one pooled sample per locality, the sampling design did not allow assessment of within-population variability or separation of genetic and environmental contributions to differences among the oil profiles.

### 3.3. Isolation of Essential Oils

Immediately after collection, the fresh plant material (aerial parts of *N. cataria*) were subjected to hydrodistillation for 2.5 h using the original Clevenger-type apparatus. Yields of the obtained essential oils (%, *w*/*w*, fresh weight) are given in Table 3. The oils were separated by extraction with diethyl ether, and dried over anhydrous magnesium sulphate. The solvent was evaporated under a gentle stream of nitrogen at room temperature, and the oils were analyzed by GC-MS and GC-FID. The oils were stored in amber vials under nitrogen at −20 °C to minimize exposure to light, oxygen, and elevated temperature. The intervals between oil isolation, NL isolation, and biological testing were 3–5 months. Isolated NL was stored under controlled conditions (in amber vials under a nitrogen atmosphere at −20 °C) to prevent photo-oxidation and thermal degradation prior to biological screenings.

### 3.4. Isolation of Nepetalactone NL

Approximately 1.5 g of the crude essential oil NC_1_ was subjected to column chromatography. The column was packed with Silica Gel 60 and pre-equilibrated with *n*-hexane. Elution was performed under gradient conditions using a stepwise gradient of mixtures of *n*-hexane and EtOAc of increasing polarity (*n*-hexane:EtOAc from 100%:0% to 80%:20%). Fractions of 10 mL were collected, and the fractions showing similar TLC profiles (TLC visualization was initially performed under UV light (254 nm), followed by spraying with 50% (*w*/*w*) aqueous H_2_SO_4_ and heating) were pooled. Two of the pooled fractions were additionally purified by preparative TLC chromatography. Preparative chromatography plates were made by spreading a thick silica gel slurry (silica:distilled water = 1:2 (*w*/*v*)) onto a 20 cm × 20 cm glass plate using a spreader, air-dried, and activated in an oven at 120 °C. As a mobile phase, a mixture of *n*-hexane:EtOAc = 90%:10% (*v*/*v*) was used. The band of interest was scored with a scalpel, and loose silica was cleared and collected from the glass backing. To elute the compound from the silica following zone extraction, the silica was transferred to a beaker and stirred with EtOAc. The suspension was filtered to extract the compound; the resulting filtrate was concentrated in vacuo, removing the residual traces of solvent under a gentle stream of nitrogen. This final step resulted in isolation of a single component (150 mg) which was subjected to NMR analysis (see the Supplementary Material).

NC_2_ and NC_3_ were also subjected to chromatographic fractionation using procedures analogous to those described for NC_1_, with the aim of isolating additional nepetalactone diastereomers. These attempts did not yield any additional isolated diastereomer. The NMR-characterized NL obtained from NC_1_ was used as the reference compound for subsequent chromatographic comparisons and co-injection experiments with all three essential oils.

### 3.5. GC-FID and GC-MS Analyses

Gas chromatography/mass spectrometry (GC-MS) analyses of *N. cataria* oil samples (NC_1_–NC_3_) were repeated three times for each sample using a Hewlett-Packard 6890N gas chromatograph equipped with a fused silica capillary column DB-5MS (5% polydiphenylsiloxane, 95% polydimethylsiloxane, 30 m × 0.25 mm, film thickness 0.25 μm; Agilent Technologies, Santa Clara, CA, USA) and coupled with a 5975B mass-selective detector from the same company. The injector and interface were operated at 250 and 300 °C, respectively. The oven temperature was raised from 70 to 290 °C at a heating rate of 5 °C/min and then isothermally held for 10 min. Helium was used as the carrier gas at a flow rate of 1.0 mL/min. The samples, 1 μL of the sample solutions in diethyl ether (1:100), were injected in a pulsed split mode (the flow was 1.5 mL/min for the first 0.5 min and then set to 1.0 mL/min throughout the remainder of the analysis; split ratio 40:1). A mass-selective detector was operated at the ionization energy of 70 eV, in the 35–500 amu range and with a scanning speed of 0.34 s. Gas chromatography (FID) analyses were carried out under the same experimental conditions and using the same column as described for the GC-MS. Relative percentages were calculated by normalization of GC-FID peak areas in the unfractionated oil samples without added reference compound; co-injection experiments were used to verify peak identity.

The constituents of the essential oils were identified by comparing their linear retention indices (relative to C_7_–C_40_ *n*-alkanes on the DB-5 MS column [47]) with values reported in the literature [48], and by matching their mass spectra with those of authentic standards as well as those from Wiley 6, NIST11, Mass-Finder 2.3 [49], along with a homemade MS library containing spectra of pure substances and known essential-oil components and, wherever possible, by co-injection with an authentic sample (alkanes, some terpenoids, and aromatics, see Table 3). The results of GC and GC-MS analyses are given in Table 3.

All three essential oils (NC_1_–NC_3_) were reanalyzed by GC-FID and GC-MS following the chromatographic fractionation experiments. The assignment of NL in each oil was verified by comparison of retention data and EI mass spectra with those of the NMR-characterized compound isolated from NC_1_ and by co-injection of each oil with this reference compound. The composition reported in Table 3 reflects these repeated analyses and the resulting reassessment of the constituent assignments. Components A, D, and E remain provisionally annotated as nepetalactone-related constituents, without assignment of specific configurations. The analyses were performed on an achiral column and did not establish absolute configuration or enantiomeric composition.

Gas chromatography/mass spectrometry of nepetalactone stereoisomer (NL) was performed on a 7890/7000B GC-MS-MS triple quadrupole system (Agilent Technologies, USA, equipped with a Combi PAL auto sampler). The fused silica capillary column DB-5MS (5% phenylmethylsiloxane, 30 m × 0.25 mm, film thickness 0.25 μm, Agilent Technologies, Palo Alto, CA, USA) was used. The injector and interface were operated at 250 °C and 325 °C, respectively. The oven temperature program was held at 50 °C for 2.25 min isothermally and then was raised to 290 °C, at a heating rate of 4 °C/min. The carrier gas was helium with a flow of 1.0 mL/min. For the NL sample, 1 mg of pure compound (NL) was dissolved in 1 mL of methanol, and 1 μL of the corresponding methanol solution was injected in a pulsed split mode (the flow was 1.5 mL/min for the first 0.5 min and then set to 1.0 mL/min throughout the remainder of the analysis; split ratio 40:1). A mass-selective detector was operated at the ionization energy of 70 eV, with acquisition mass range 50–650 and scan time 0.32 s. Semi-quantitative analysis was carried out directly from the peak area in the GC profile. Linear retention indices (RIs) were determined based on the retention times of C_7_–C_40_ *n*-alkanes on the DB-5MS column, using the above-mentioned temperature programs.

Relative standard deviation (RSD) of peak areas of the repeated measurements (independent sample preparations and GC-MS) was for all substances below 1%. In repeated measurements, no significant deviation of the component retention times was observed.

### 3.6. NMR Analysis

^1^H and ^13^C NMR spectra of the isolated nepetalactone were recorded on a Bruker Avance III 400 MHz NMR spectrometer (Bruker, Fällanden, Switzerland), operating at 400 MHz (^1^H) and at 100.6 MHz (^13^C), equipped with a 5 mm dual ^13^C/^1^H probe head. All NMR spectra were recorded at 20 °C. The spectra were recorded in chloroform-d, with tetramethylsilane (TMS) serving as the internal standard. Chemical shifts were reported on a δ (ppm) scale and referenced to TMS (δ_H_ = 0.00 ppm) in ^1^H and to the (residual) solvent signal in ^13^C NMR and heteronuclear 2D spectra (residual CHCl_3_ δ_H_ = 7.26 ppm and ^13^CDCl_3_ δ_C_ = 77.16 ppm). Scalar coupling constants (*J*) are reported in Hertz (Hz). Standard Bruker pulse sequences were used for acquiring both 1D and 2D NMR spectra. The sample (20 mg of isolated nepetalactone) was dissolved in 1 mL of CDCl_3_, and 0.7 mL of the solution transferred into a 5 mm NMR tube Wilmad 528-TR-7 (SP Wilmad-Lab Glass, Vineland, NJ, USA).

#### ^1^H NMR Full-Spin Analysis

The comprehensive ^1^H NMR spin analysis for the isolated compound (NL) was conducted by manually refining δ_H_ and *J* values to match the experimentally obtained data, subsequently enhanced through optimization using MestReNova 11.0.3 software (tools/spin simulation). This approach [50] enabled a systematic adjustment of all calculated NMR parameters, finally reaching good agreement between simulated and experimental data (NRMSD < 0.05%) for the isolated compound.

### 3.7. Biological Activity Assays

#### 3.7.1. Lipid Peroxidation Inhibition by Thiobarbituric-Acid–Malondialdehyde Test (TBA–MDA Assay)

The thiobarbituric-acid–malondialdehyde test (TBA–MDA assay) is a widely utilized spectrophotometric assay for assessing lipid peroxidation. Samples containing lipids are heated with TBA where malondialdehyde (MDA), a reactive secondary LP product, at low pH, allows the formation of pink-colored MDA–(TBA)_2_ adduct, which displays strong absorbance at 530 nm [51]. In the presence of examined samples (*N. cataria* oils: NC_1_–NC_3_ and isolated NL), LP and its inhibition were measured using the TBA–MDA test. The experimental procedure for assessing inhibition of AAPH-initiated lipid oxidation using the TBA–MDA endpoint was essentially carried out according to the procedure described by Brizzolari et al. [42]. Absorbances were read on a Thermo Scientific Evolution 60S UV–vis spectrophotometer (Madison, WI, USA).

The absorbance of the MDA–(TBA)_2_ adduct in the supernatant, read at 530 nm, was used to calculate the inhibition percentage of LP using the following equation:

Inhibition of lipid peroxidation (%) = 100 × (A_c_ − A_s_)/(A_c_ − A_b_)

where A_c_ is the absorbance of the control (methanol solution of PL90) which is treated with the AAPH and TBA solution; A_s_ is the absorbance of the sample (methanol solution of PL90 containing tested sample(s)) which is treated with the AAPH and TBA solution; and A_b_ is the absorbance of the blank ((methanol solution of PL90 not treated by AAPH, but treated with TBA solution (monitoring MDA level in the lipid before LP initiation by AAPH)).

Samples were evaluated for LP-inhibitory activity at 1000 mg/L in the final reaction mixture, and those demonstrating inhibition exceeding 50% were tested across a broader concentration range to ascertain IC_50_ values. The IC_50_ values represent the concentrations of NC_1_–NC_3_ and NL samples studied in the reaction mixture which induce a 50% LP-inhibition effect following 3 h of incubation. The experiment was designed so that methanol concentration was set constant in all assay mixtures for which the absorbances were read. Following the identical procedure, well-established antioxidants caffeic acid and Trolox were used as reference points, assayed at concentrations of 10 mg/L (caffeic acid) and 25 mg/L (Trolox) in the final reaction mixture. Each experiment was conducted in technical triplicate (three parallel measurements per concentration within the same experiment). The obtained results were plotted; IC_50_ values were calculated and are reported in Table 4 and in Supplementary Material Figure S8.

#### 3.7.2. Concentration-Dependent Effects of *Nepeta cataria* Oils and NL on Micronucleus Frequency in Cultured Human Lymphocytes

The concentration-dependent effects on micronucleus frequency in cultured human lymphocytes of the examined samples was assessed in vitro, using one of the most well-validated techniques for measuring DNA damage, chromosome instability, and cell death: the cytokinesis-block micronucleus assay (CBMN). Micronucleus expression in peripheral blood lymphocytes is well-established as a method, extensively used as a valuable genotoxic endpoint to assess cytogenetic damage in human population. For micronucleus preparation, the cytokinesis-block method (CBMN) of Fenech and Morley [43,46], with certain modifications as outlined by Stanković et al. [9], was used. A minimum of 1000 binucleated (BN) cells per sample were scored, and micronuclei were identified based on the standards set by Countryman and Heddle [52], along with Fenech and Morley [43,46].

Amifostine WR-2721, a cytoprotective thiol prodrug that can be transformed into an active sulfhydryl compound that scavenges radiation-induced free radicals, neutralizes the reactive metabolites of alkylating agents, and prevents cell damage, and alkylating and interstrand cross-linking agent mitomycin C (MMC) [53], capable of inducing chromosome aberrations in vitro [45,54], DNA damage [9], and MN formation, were used as controls. One untreated cell culture served as a baseline control (blank).

##### Subjects

Blood samples used in this study were obtained from healthy adult male volunteers who provided explicit, written informed consent prior to participation. To ensure participant privacy and data integrity, all collections were entirely voluntary, completely anonymized, and coded upon receipt. Blood drawing was performed by a qualified medical professional using standard sterile venipuncture techniques. All biological assays and sample processing were conducted at the Nuclear Facilities of Serbia, Vinča, which performs certified and accredited genotoxicity analyses routinely used for the biological monitoring of personnel in high-risk occupational environments.

Four healthy, non-smoking male volunteers (average age 34 years), who had not been exposed to chemicals, drugs, or other substances that could affect micronucleus frequencies, consented to the use of their blood in the experiment. A safety protocol regarding blood-borne pathogens and/or biohazard was applied. The study was conducted in accordance with the institutional safety standards and the ethical principles outlined in the World Medical Association’s Declaration of Helsinki [55]. The blood samples were obtained at the Medical Unit, following the current Health and Ethical guidelines in Serbia (Health Care Law, 2019) [56].

##### Sample Collection

Peripheral blood (PB) samples (two 5 mL aliquots of blood from each subject (donor)) were collected under sterile conditions and processed according to the SRPS ISO 17099:24 recommendations [57]. A certified, trained medical laboratory technician drew PB samples by venipuncture using a sterile syringe; the samples were then transferred into heparinized, sterile vacutainer tubes (Becton Dickinson, Bradford, MA, USA) and mixed thoroughly. To prevent possible bias and ensure the confidentiality of donors, all samples were coded and stored at 4 °C, aseptically transported to the lab immediately, and processed within 3 h of sampling on the same day.

##### Lymphocyte Cultures for Cytokinesis-Block Micronucleus (CBMN) Assay

Lymphocyte cultures were initiated immediately by adding 0.5 mL of the heparinized whole blood (containing approximately 2 × 10^6^ leukocytes) to 4.5 mL Gibco^TM^ PB-MAX^TM^ Karyotyping Medium (an optimized RPMI 1640 medium, supplemented with 15% fetal bovine serum, L-glutamine, and 2.4 µg/mL phytohemagglutinin (PHA), developed for the short-term culture of peripheral blood lymphocytes for cytogenetic studies). The lymphocytes were incubated in a thermostat (BOV-H50, BIOBASE, Jinan, Shandong, China) at 37 °C. After 44 h of incubation, cytochalasin B at a final concentration of 6 µg/mL was added to block cytokinesis, and lymphocyte cultures were reincubated for a further 28 h. At 72 h post-PHA-stimulation, the cultures were washed with 0.9% NaCl and harvested through centrifugation at 1200 rpm for 8 min. Cells were treated with hypotonic solution (0.56% KCl:0.9% NaCl = 1:1 (*v*/*v*)) at 4 °C for 3 min, spun again, and prefixed in Carnoy’s fixative utilizing methanol (methanol/acetic acid = 3:1 (*v*/*v*)) with gentle shaking. This fixation procedure was repeated thrice by centrifugation at 500 rpm for 5 min, and finally, the cells were resuspended in the fixative and dropped onto prechilled slides [46]. The slides were air-dried and stained with alkaline Giemsa stain (2%). Visual scoring using the Olympus CX31 Binocular Microscope (Evident Scientific, Hamburg, Germany) was performed to analyze the binucleated (BN) cells (×40 and ×100 magnification). To minimize variability, all the coded slides were scored blindly by a single scorer. To determine the frequency of BNMN, at least 1000 binucleated (BN) cells with well-preserved cytoplasm were scored per subject on the coded slides. The scoring criteria for binucleated lymphocytes with micronuclei (BNMN) evaluation adhered to the criteria established by Countryman and Heddle [52] and Fenech et al. [58]. A culture prepared in this way served as a baseline control (untreated culture, or blank).

##### In Vitro Cytokinesis-Block Micronucleus (CBMN) Assay of *Nepeta cataria* Oils and of Nepetalactone NL

The lymphocyte cultures were treated with three concentrations (1, 2, and 4 μg/mL) of the tested samples (NC_1_–NC_3_ and NL), added 1 h after initiating the culture stimulation. The samples were tested at 1, 2, and 4 µg/mL for an exploratory assessment of their effects on MN frequency and proliferative progression. The results do not establish a non-toxic exposure range. Treatment with the studied samples lasted for 19 h, after which all cultures were rinsed with pure medium, transferred into 5 mL fresh Gibco PB-MAX Karyotyping Medium, and incubated for an additional 24 h. After 44 h of incubation, cytochalasin B at a final concentration of 6 µg/mL was added to block cytokinesis, and lymphocyte cultures were reincubated for a further 28 h. At 72 h post-PHA-stimulation, the workflow aligned with the instructions described in Section “Lymphocyte Cultures for Cytokinesis-Block Micronucleus (CBMN) Assay”.

Two separate lymphocyte cultures were also used, one treated only with the mutagenic agent and micronucleus inducer MMC (0.2 µg/mL, in phosphate buffer), and another with reference cytoprotective agent amifostine WR-2721 (1 µg/mL). Agent (MMC or amifostine WR-2721) was added to the culture 25 h after PHA stimulation and left until harvest. Upon the addition, cultures were incubated at 37 °C. After 44 h of incubation, cytochalasin B was added to block cytokinesis, and lymphocyte cultures were reincubated for a further 28 h, following the methodology outlined in Section “Lymphocyte Cultures for Cytokinesis-Block Micronucleus (CBMN) Assay”. The potential effects of the studied samples were also assessed based on the activity of these two agents (Table 5).

In addition, the impact of the reduction of MN frequency on cell proliferation of the studied samples (NC_1_–NC_3_ and NL) was estimated by the cytokinesis-block proliferation index (CBPI), calculated as proposed by Surralles et al. [53]. The cytokinesis-block proliferation index (CBPI) was calculated using the formula: CBPI = [MI + 2MII + 3(MIII + MIV)]/N, where MI through MIV represent the number of cells containing one to four nuclei, respectively, and N is the total number of cells scored. Both three-nucleated (MIII) and tetra-nucleated (MIV) cells were equally considered to be in their third cell cycle. For the analysis of MN, only binucleated cells with well-preserved cytoplasm were scored using a light microscope (Olympus CX31), with a ×40 and ×100 magnification. The criteria adhered to for selection of binucleated cells and identification of MN were those of Fenech et al. [58]. The numbers of BN cells with one, two, three, or more MN were then classified. The data for each treatment were expressed as the frequency of MN per 1000 binucleated cells.

#### 3.7.3. Statistics and Index Calculation

The LP inhibition concentration–response curves were modeled via nonlinear regression using a four-parameter logistic (4-PL) function. Statistical analyses and graphical data processing were performed using the free statistical software R version 4.5.3. in the RStudio environment (version 2024.09.0+375). All packages used are open-source and freely available for non-commercial use (GNU GPL license). All curve-fitting procedures and subsequent IC_50_ estimations were performed using the Dose–Response Curves (drc) package in R (version 4.5.3). For each compound, three independent experiments were performed, each in technical triplicate. Technical replicates were averaged prior to curve fitting. IC_50_ values were calculated from the fitted model, reported with standard error (SE) and 95% confidence intervals (95% CI) derived from the model, computed using the delta method. Statistical analysis of the CMBN data was performed using Origin software package version 7.0. The statistical significance of difference between the data pairs was evaluated by analysis of variance (one-way ANOVA), followed by the Tukey test. Differences were considered statistically significant at *p* < 0.01. The results (index calculation) are presented as the percentage of change compared to the controls.

## 4. Conclusions

The three pooled *N. cataria* samples from southern and southeastern Serbia showed differences in the relative abundances of their major oil constituents. NC_1_ and NC_3_ were dominated by the same assigned relative nepetalactone isomer, *trans*-*cis*-nepetalactone (4a*α*,7*α*,7a*β*-nepetalactone; NL), whereas NC_2_ contained a larger proportion of nepetalactone-related components D and E. The constitution and relative configuration of NL isolated from NC_1_ were established by NMR spectroscopy and full-spin analysis. The configurations of A, D, and E remain unresolved. Because one pooled sample was examined per locality, the results do not establish stable population chemotypes or distinguish genetic from environmental contributions to compositional variation.

The oils and isolated NL reduced the TBA-reactive signal in the AAPH/phospholipid model and were associated with concentration-dependent differences in micronucleus frequency in cultured human lymphocytes. Mean micronucleus frequencies at 4 µg/mL were higher than the untreated-control mean for all four samples (NC_1_–NC_3_ and NL). The oil IC_50_ estimates were imprecise, and the four-donor cellular experiment remains exploratory. The contribution of NL to the whole-oil responses and the mechanism underlying the lymphocyte findings were not established. Further structural characterization of the remaining major components and studies incorporating complementary cytotoxicity endpoints are needed to clarify these observations.

## Figures and Tables

**Table 1:** *Nepeta cataria* L. investigated samples with provenance, abbreviations, and voucher specimens.

Sample Label	Locality	Coordinates	Voucher Specimen
NC_1_	Rudina, Izvor, Bosilegrad, SE Serbia	42°30′ N 22°30′ E	HMN18955
NC_2_	Niš, S Serbia	43°19′29″ N 21°54′11″ E	HMN18956
NC_3_	Buštranje, Pčinja District, S Serbia	42°19′50″ N 21°45′18″ E	HMN18957

**Table 2:** ^1^H (400 MHz) and ^13^C (100.6 MHz) NMR spectral data (in CDCl_3_) of the isolated NL (NMR parameters are derived from manual iterative full-spin analysis).

Position	δ_H_ (m, *J* (Hz), Integral) ^a^	δ_C_ (m, *J* (Hz)) ^b^	gHMBC ^c^	NOESY ^d^
1	/	170.33 (td, ^2^*J*_H-7A,C-1_ = 7.2, ^3^*J*_H-3,C-1_ = 4.3)	/	/
3	6.24 (dq, ^4^*J*_3,4A_ = 2.9, ^4^*J*_3,8_ = 1.7, 1 H)	135.93 (dqd, ^1^*J*_H-3,C-3_ = 195.5, ^3^*J*_H-8,C-3_ = 7.6, ^3^*J*_H-4A,C-3_ = 4.3)	1, 4, 4A, 8	8
4	/	120.54 (m)	/	/
4A	2.69 (ddddqdd, ^3^*J*_4A,7A_ = 15.1, ^3^*J*_4A,5a_ = 11.7, ^3^*J*_4A,5b_ = 7.2, ^4^*J*_3,4A_ = 2.9, ^4^*J*_4A,8_ = 1.5, ^4^*J*_4A,6a_ = ^4^*J*_4A,6b_ = 0.1, 1 H)	37.39 (m ^e^, ^1^*J*_H-4A,C-4A_ = 125.8)	1, 3, 4, 5, 7	9
5a	1.37 (dddd, ^2^*J*_5a,5b_ = 12.6, ^3^*J*_4A,5a_ = 11.7, ^3^*J*_5a,6b_ = 10.7, ^3^*J*_5a,6a_ = 8.5, 1 H)	26.21 (ddsext, ^1^*J*_H-5a,C-5_ = 133.4, ^1^*J*_H-5b,C-5_ = 129.1, ^2^*J*_H-4A,C-5_ = ^2^*J*_H-6a,C-5_ = ^2^*J*_H-6b,C-5_ = ^3^*J*_H-7,C-5_ = ^3^*J*_H-7A,C-5_ = 4.0)	4, 4A, 6	7A
5b	1.96 (dddd, ^2^*J*_5a,5b_ = 12.6, ^3^*J*_5b,6a_ = 8.8, ^3^*J*_4A,5b_ = 7.2, ^3^*J*_5b,6b_ = 2.8, 1 H)		4A, 6, 7, 7A	/
6a	1.38 (dddddd, ^2^*J*_6a,6b_ = 13.6, ^3^*J*_5b,6a_ = 8.8, ^3^*J*_5a,6a_ = 8.5, ^3^*J*_6a,7_ = 2.7, ^4^*J*_6a,7A_ = 0.3, ^4^*J*_4A,6a_ = 0.1, 1 H)	32.15 (m ^e^, ^1^*J*_H-6a,C-6_ = ^1^*J*_H-6b,C-6_ = 130.7)	4A, 5, 7A, 9	/
6b	2.15 (dddddd, ^2^*J*_6a,6b_ = 13.6, ^3^*J*_5a,6b_ = 10.7, ^3^*J*_6b,7_ = 8.4, ^3^*J*_5b,6b_ = 2.8, ^4^*J*_6b,7A_ = 0.3, ^4^*J*_4A,6b_ = 0.1, 1 H)		4A, 5, 6, 7, 7A, 9	/
7	2.50 (dqdd, ^3^*J*_6b,7_ = 8.4, ^3^*J*_7,9_ = 7.1, ^3^*J*_7,7A_ = 6.8, ^3^*J*_6a,7_ = 2.7, 1 H)	30.05 (m ^e^, ^1^*J*_H-7,C-7_ = 136.0, ^2^*J*_H-9,C-7_ = 4.7)	4A, 5, 6, 7A, 9	9
7A	2.35 (ddddq, ^3^*J*_4A,7A_ = 15.1, ^3^*J*_7,7A_ = 6.8, ^4^*J*_6a,7A_ = ^4^*J*_6b,7A_ = ^4^*J*_7A,9_ = 0.3, 1 H)	49.16 (m ^e^, ^1^*J*_H-7A,C-7A_ = 122.6)	1, 4, 4A, 5, 7, 9	5a
8	1.72 (dd, ^4^*J*_3,8_ = 1.7, ^4^*J*_4A,8_ = 1.5, 3 H)	14.38 (qd, ^1^*J*_H-8,C-8_ = 127.7, ^3^*J*_H-3,C-8_ = 3.6)	3, 4, 4A,	3
9	1.12 (dd, ^3^*J*_7,9_ = 7.1, ^4^*J*_7A,9_ = 0.3, 3 H)	17.70 (qdtd, ^1^*J*_H-9,C-9_ = 125.8, ^3^*J*_H-7A,C-9_ = 7.8, ^3^*J*_H-6a,C-9_ = ^2^*J*_H-7,C-9_ = 5.6, ^3^*J*_H-6b,C-9_ = 3.0)	6, 7, 7A	4A

^a^ Proton chemical shifts (δ_H_) are reported to two decimal places with coupling constants (*J*) presented as positive absolute values; the full multi-decimal precision and mathematically signed parameters are retained in Table S1 (Supporting Information) for lineshape simulation reproduction. ^b^ ^1^H–^13^C coupling constants. Long-range (^2^*J* and ^3^*J*) coupling constants were tentatively assigned based on expected magnitude ranges; ^3^*J* values were correlated with dihedral angles estimated from the 3D model as supporting evidence. ^c^ gHMBC correlations observed between the hydrogen in this row and the carbon in the listed position. ^d^ Key NOESY interactions are included to verify the relative configuration and stereocenter assignment within the rigid bicyclic ring system; a comprehensive list of all observed spatial correlations is provided in Table S1. ^e^ The main resolved coupling constants are reported, whereas the remaining couplings are unresolved.

**Table 4:** Lipid peroxidation inhibition effects of *N. cataria* oils (NC_1_–NC_3_), pure NL, and of selected antioxidant standards.

Sample	IC_50_ (Fitted, mg/L) ^a^	SE (mg/L) ^b^	95% CI (mg/L) ^c^	R^2 d^	IC_50_ (μM) ^e^
NC_1_	102.5	174.4	n.d. ^f^	0.973	n.a. ^g^
NC_2_	272.1	185.4	n.d.	0.993	n.a.
NC_3_	160.3	278.8	n.d.	0.989	n.a.
NL	23.9	0.5	22.7–25.2	0.9994	143.8
Caffeic acid	3.0	0.4	2.1–4.0	0.9995	16.7
Trolox	6.3	0.3	5.6–6.9	0.9991	25.2

^a^ the IC_50_ values were estimated using a 4-PL model; ^b^ standard error of the 4-PL estimate; ^c^ the acceptable margin of error for the results was expressed as a 95% confidence interval; ^d^ coefficient of determination for the 4-PL model, used to assess the quality of the fit; ^e^ for pure NL and for antioxidants used as a positive control, the IC_50_ values were recalculated and also expressed as μM; ^f^ n.d., not reliably determined. This limitation arose because the nonlinear regression model could not mathematically converge to generate stable intervals, owing to high standard errors, complex oil concentration–response data variability, and a limited number of data points around the IC_50_ threshold; ^g^ n.a., not applicable.

**Table 5:** Effects of *N. cataria* essential oils (NC_1_–NC_3_) and isolated nepetalactone (NL) on micronucleus frequency and proliferation index (CBPI) in cultured human lymphocytes.

Concentration (µg/mL)	MN per 1000 BN Cells ± SD	BN Cells with MN (%) ± SD	MN per Micronucleated BN Cell ± SD	CBPI ± SD	MN Frequency
Blank control	22.15 ± 1.19	1.84 ± 0.16	1.20 ± 0.09	1.47 ± 0.13	100.0%
Amifostine–WR 2721—1.0 µg/mL	18.25 ± 1.58 ^a^	1.51 ± 0.12	1.20 ± 0.03	1.55 ± 0.16	82.4%
MMC—0.2 µg/mL	32.50 ± 2.59 ^a,b^	2.79 ± 0.24	1.17 ± 0.04	1.72 ± 0.09	146.7%
NC_1_—1 µg/mL	15.36 ± 1.85 ^a,b,c^	1.34 ± 0.09	1.14 ± 0.08	1.51 ± 0.13	69.3%
NC_1_—2 µg/mL	17.02 ± 0.95 ^a,c^	1.48 ± 0.20	1.15 ± 0.15	1.51 ± 0.08	76.8%
NC_1_—4 µg/mL	24.81 ± 1.19 ^a,b,c^	1.91 ± 0.12	1.31 ± 0.06	1.65 ± 0.17	112.0%
NC_2_—1 µg/mL	16.12 ± 1.32 ^a,b,c^	1.42 ± 0.24	1.14 ± 0.12	1.56 ± 0.09	72.8%
NC_2_—2 µg/mL	18.33 ± 0.99 ^a,c^	1.48 ± 0.06	1.24 ± 0.03	1.74 ± 0.26	82.7%
NC_2_—4 µg/mL	23.83 ± 2.25 ^b,c^	1.85 ± 0.21	1.29 ± 0.08	1.76 ± 0.11	107.6%
NC_3_—1 µg/mL	17.84 ± 1.26 ^a,b,c^	1.45 ± 0.11	1.23 ± 0.10	1.71 ± 0.18	80.5%
NC_3_—2 µg/mL	15.88 ± 1.01 ^a,b,c^	1.40 ± 0.10	1.14 ± 0.04	1.65 ± 0.05	71.7%
NC_3_—4 µg/mL	24.55 ± 1.65 ^a,b,c^	1.83 ± 0.21	1.18 ± 0.07	2.01 ± 0.65	110.8%
NL—1 µg/mL	17.30 ± 0.91 ^a,b,c^	1.45 ± 0.10	1.17 ± 0.08	1.67 ± 0.04	78.1%
NL—2 µg/mL	22.01 ± 1.05 ^b,c^	1.86 ± 0.05	1.18 ± 0.06	1.71 ± 0.10	99.4%
NL—4 µg/mL	28.46 ± 1.11 ^a,b,c^	2.41 ± 0.13	1.20 ± 0.06	1.65 ± 0.10	128.5%

Values are expressed as means ± SD calculated across four individual donors, utilizing an exact denominator of 1000 binucleated cells per donor (totaling 4000 scored cells per treatment group). MN per 1000 BN cells denotes the total micronucleus count normalized to 1000 scored binucleated cells examined per donor for each concentration; BN cells with MN (%) denotes the percentage of binucleated cells containing at least one micronucleus; MN per micronucleated BN cell represents the total micronucleus count divided by the number of micronucleated binucleated cells; MN frequency (% of untreated control) indicates the incidence of micronuclei in binucleated cells expressed relative to the control group, calculated as 100 × (mean donor micronucleus frequency/mean untreated control frequency); CBPI denotes the cytokinesis-block proliferation index. The statistical significance of differences was evaluated by one-way analysis of variance (ANOVA) followed by Tukey’s post hoc test. A difference was considered statistically significant at *p* < 0.01. ^a^ Statistically significant difference compared with the untreated control group (*p* < 0.01); ^b^ statistically significant difference compared with amifostine (WR-2721) (*p* < 0.01); ^c^ statistically significant difference compared with mitomycin C (MMC) (*p* < 0.01).

## Data Availability

The datasets and materials generated and/or analyzed during the current study are available from the corresponding author on reasonable request.

## Supplementary Materials

The following supporting information can be downloaded at: https://www.mdpi.com/article/10.3390/plants15213406/s1, Figures S1–S8, Figure S1. Total ion chromatograms (TICs) of *N. cataria* essential oil samples; MS scans of the iridoids (MI) identified/detected in *N. cataria* oils; Figure S2. Total ion chromatogram (TIC) of the isolated NL; MS scan of isolated NL; Table S1. ^1^H (400 MHz) and ^13^C (100.6 MHz) NMR spectral data (in CDCl_3_) of the isolated NL (NMR parameters derived from manual iterative full-spin analysis); Figure S3. ^1^H NMR (400 MHz, CDCl_3_) spectrum of the isolated NL; Figure S4. Comparison of the simulated and observed ^1^H NMR (400 MHz, CDCl_3_) NL spectrum; Figure S5. Selected observed ^1^H–^13^C coupling constants represented on 3D model of the NL; Figure S6. ^13^C NMR (100.6 MHz, CDCl_3_) spectrum of the isolated NL; Figure S7. Comparison of the simulated and observed coupled ^13^C (100.6 MHz, CDCl_3_) NMR spectrum of selected carbon signals of NL; Figure S8. LP-inhibitory activity (IC_50_, mg/L) of *N. cataria* oil samples NC_1_–NC_3_, isolated NL, and standards (caffeic acid and Trolox).

## References

[1] Yu Y., Yan Y., Niu F., Wang Y., Chen X., Su G., Liu Y., Zhao X., Qian L., Liu P. (2021). Ferroptosis: A cell death connecting oxidative stress, inflammation and cardiovascular diseases. Cell Death Discov..

[2] Valgimigli L. (2023). Lipid peroxidation and antioxidant protection. Biomolecules.

[3] Grotto D., Maria L.S., Valentini J., Paniz C., Schmitt G., Garcia S.C., Pomblum V.J., Rocha J.B.T., Farina M. (2009). Importance of the lipid peroxidation biomarkers and methodological aspects for malondialdehyde quantification. Quím. Nova.

[4] Ayna A., Caglayan C., Taysi S. (2025). Cellular and molecular mechanisms of oxidative DNA damage and repair. Medicina.

[5] Poetsch A.R. (2020). The genomics of oxidative DNA damage, repair, and resulting mutagenesis. Comput. Struct. Biotechnol. J..

[6] Fenech M., Knasmueller S., Bolognesi C., Holland N., Bonassi S., Kirsch-Volders M. (2020). Micronuclei as biomarkers of DNA damage, aneuploidy, inducers of chromosomal hypermutation and as sources of pro-inflammatory DNA in humans. Mutat. Res. Rev. Mutat. Res..

[7] Fenech M. (2020). Cytokinesis-block micronucleus cytome assay evolution into a more comprehensive method to measure chromosomal instability. Genes.

[8] Bonassi S., El-Zein R., Bolognesi C., Fenech M. (2011). Micronuclei frequency in pe-ripheral blood lymphocytes and cancer risk: Evidence from human studies. Mutagenesis.

[9] Stanković M., Tesević V., Vajs V., Todorović N., Milosavljević S., Godevac D. (2008). Antioxidant properties of grape seed extract on human lymphocyte oxidative defence. Planta Med..

[10] Rajković K., Stanković M., Markićević M., Zavisić G., Vranješ Đurić S., Janković D., Obradović Z., Stanković D. (2024). Chemical composition and protective possibilities of *Juglans nigra* leaves and green husks extracts: DNA binding and micronucleus assay in human lymphocytes. Plants.

[11] Chandimali N., Bak S.G., Park E.H., Lim H.-J., Won Y.-S., Kim E.-K., Park S.-I., Lee S.J. (2025). Free radicals and their impact on health and antioxidant defenses: A review. Cell Death Discov..

[12] Huang Y., Ebrahimi H., Berselli E., Foti M.C., Amorati R. (2025). Essential oils as antioxidants: Mechanistic insights from radical scavenging to redox signaling. Antioxidants.

[13] Formisano C., Rigano D., Senatore F. (2011). Chemical constituents and biological activities of *Nepeta* species. Chem. Biodivers..

[14] Süntar I., Nabavi S.M., Barreca D., Fischer N., Efferth T. (2018). Pharmacological and chemical features of *Nepeta* L. genus: Its importance as a therapeutic agent. Phytother. Res..

[15] Ali K., Ahmed S., Ali A., Dong X., Liu C., Fatima B., Law D., Hussain M., Ali I. (2026). Phytochemistry and pharmacological properties of the genus *Nepeta* documented between 2021 and 2025. ChemistrySelect.

[16] Sharma A., Nayik G.A., Cannoo D.S., Ozturk M., Hakeem K. (2019). Pharmacology and toxicology of *Nepeta cataria* (catmint) species of genus *Nepeta*: A review. Plant and Human Health.

[17] Dmitrović S., Nestorović Živković J., Smailagić D., Trajković M., Banjac N., Ninković S., Stanišić M. (2025). Via air or rhizosphere: The phytotoxicity of *Nepeta* essential oils and malus dihydrochalcones. Plants.

[18] Liblikas I., Santangelo E.M., Sandell J., Baeckström P., Svensson M., Jacobsson U., Unelius C.R. (2005). Simplified isolation procedure and interconversion of the diastereomers of nepetalactone and nepetalactol. J. Nat. Prod..

[19] Banjanac T., Milutinović M., Matekalo D., Popović N., Petrović L., Gašić U., Skorić M., Šiler B., Lukić T., Stupar A. (2026). Heterogeneity of iridoid biosynthesis in catmints: Molecular background in a phylogenetic context. J. Integr. Plant Biol..

[20] Tay N.E., Du B., Gillam T.A., Thomas M.L., Algar D., Fleming P.A. (2026). Feral cat responses to silver vine: Implications for feral cat management. J. Chem. Ecol..

[21] Aćimović M., Zeremski T., Kiprovski B., Brdar-Jokanović M., Popović V., Koren A., Sikora V. (2021). *Nepeta cataria*—Cultivation, chemical composition and biological activity. J. Agron. Technol. Eng. Manag. (JATEM).

[22] Velasco Negueruela A., Mata Rico M., Bermejo Benito P., Pérez-Alonso M.J. (1988). Composicion de los aceites esenciales de *Nepeta nepetella* subsp. *aragonensis*, *Nepeta coerulea* subsp. *coerulea* y *Nepeta cataria*. Giorn. Bot. Ital..

[23] Schultz G., Simbro E., Belden J., Zhu J., Coats J. (2004). Catnip, *Nepeta cataria* (Lamiales: Lamiaceae)—A closer look: Seasonal occurrence of nepetalactone isomers and comparative repellency of three terpenoids to insects. Environ. Entomol..

[24] Nestorović Živković J.M., Živković S., Šiler B., Aničić N., Dmitrović S., Divac Rankov A., Giba Z., Mišić D. (2018). Differences in bioactivity of three endemic *Nepeta* species arising from main terpenoid and phenolic constituents. Arch. Biol. Sci..

[25] Gokce I. (2010). Antioxidant capacity of Catnip (*Nepeta cataria*). Asian J. Chem..

[26] Adiguzel A., Ozer H., Sokmen M., Gulluce M., Sokmen A., Kilic H., Sahin F., Baris O. (2009). Antimicrobial and antioxidant activity of the essential oil and methanol extract of *Nepeta cataria*. Pol. J. Microbiol..

[27] Ashrafi B., Ramak P., Ezatpour B., Talei G.R. (2019). Biological activity and chemical composition of the essential oil of *Nepeta cataria* L. J. Res. Pharm..

[28] Gkinis G., Bozin B., Mimica-Dukić N., Tzakou O. (2010). Antioxidant activity of *Nepeta nuda* L. ssp. *nuda* essential oil rich in nepetalactones from Greece. J. Med. Food.

[29] Mollova S., Dzhurmanski A., Fidan H., Bojilov D., Manolov S., Dincheva I., Stankov S., Stoyanova A., Ercisli S., Assouguem A. (2023). Chemical composition of essential oils from *Nepeta transcaucasica* Grossh. and *Nepeta cataria* L. cultivated in Bulgaria and their antimicrobial and antioxidant activity. ACS Omega.

[30] Aćimović M., Travičić V., Simić K., Varga A., Pezo L., Vulić J., Cabarkapa I. (2022). Chemical profile of *Nepeta cataria* L. var. *citriodora* (Becker) essential oil and in vitro evaluation of biological activities. Acta Sci. Pol. Hortorum Cultus.

[31] Joshi M., Karakoti H., Kumar R., Prakash O., Pant A., Rawat D. (2021). Chemical composition and biological activities of *Nepeta hindostana* (Roth) Haines, *Nepeta graciliflora* Benth. and *Nepeta cataria* L. from India. J. Med. Herb..

[32] Handjieva N.V., Popov S.S., Evstatieva L.N. (1996). Constituents of essential oils from *Nepeta cataria* L., *N. grandiflora* M.B. and *N. nuda* L. J. Essent. Oil Res..

[33] Radulović N., Blagojević P.D., Rabbitt K., Menezes F.d.S. (2011). Essential oil of *Nepeta* x *faassenii* Bergmans ex Stearn (*N. mussinii* Spreng. x *N. nepetella* L.): A comparison study. Nat. Prod. Commun..

[34] Liu H., Liang S., Zhu M., Shi W., Xu C., Wei W., Zhan R., Ma D. (2024). A fused hybrid enzyme of 8-hydroxygeraniol oxidoreductase (8HGO) from *Gardenia jasminoides* and iridoid synthase (ISY) from *Catharanthus roseus* significantly enhances nepetalactol and iridoid production. Planta.

[35] Hernández Lozada N.J., Hong B., Wood J.C., Caputi L., Basquin J., Chuang L., Kunert M., Rodríguez López C.E., Langley C., Zhao D. (2022). Biocatalytic routes to stereo-divergent iridoids. Nat. Commun..

[36] Scaffidi A., Algar D., Bohman B., Ghisalberti E., Flematti G. (2016). Identification of the cat attractants isodihydronepetalactone and isoiridomyrmecin from *Acalypha indica*. Aust. J. Chem..

[37] Bol S., Scaffidi A., Bunnik E.M., Flematti G.R. (2022). Behavioral differences among domestic cats in the response to cat-attracting plants and their volatile compounds reveal a potential distinct mechanism of action for actinidine. BMC Biol..

[38] Uenoyama R., Miyazaki T., Hurst J.L., Beynon R.J., Adachi M., Murooka T., Onoda I., Miyazawa Y., Katayama R., Yamashita T. (2021). The characteristic response of domestic cats to plant iridoids allows them to gain chemical defense against mosquitoes. Sci. Adv..

[39] Bol S., Caspers J., Buckingham L., Anderson-Shelton G.D., Ridgway C., Buffington C.A., Schulz S., Bunnik E.M. (2017). Responsiveness of cats (Felidae) to silver vine (*Actinidia polygama*), Tatarian honeysuckle (*Lonicera tatarica*), valerian (*Valeriana officinalis*) and catnip (*Nepeta cataria*). BMC Vet. Res..

[40] Nelson D.L. (1968). Methylcyclopentane monoterpenes of *Nepeta cataria* and *Actinidia polygama*. Doctoral Dissertation.

[41] Sakai T., Nakajima K., Sakan S. (1980). New monoterpene lactones of the iridane type from *Actinidia polygama* Miq. Bull. Chem. Soc. Jpn..

[42] Brizzolari A., Foti M.C., Saso L., Ciuffreda P., Lazarević J., Santaniello E. (2022). Evaluation of the radical scavenging activity of some representative isoprenoid and aromatic cytokinin ribosides (N^6^-substituted adenosines) by in vitro chemical assays. Nat. Prod. Res..

[43] Fenech M., Morley A.A. (1986). Cytokinesis—Block micronucleus method in human lymphocytes: Effect of in vivo ageing and low dose X—Irradiation. Mutat. Res..

[44] Fenech M. (1991). Optimization of micronucleus assay for biological dosimetry. Prog. Clin. Biol. Res..

[45] Fenech M. (2000). A mathematical model for the in vitro micronucleus assay predicts false negative results if micronuclei are not specifically scored in binucleated cells or in cells that have completed one nuclear division. Mutagenesis.

[46] Fenech M. (1993). The cytokinesis-block micronucleus technique: A detailed description of the method and its application to genotoxicity studies in human populations. Mutat. Res..

[47] Van den Dool H., Kratz P.D. (1963). A generalization of the retention index system including linear temperature programmed gas liquid partition chromatography. J. Chromatogr..

[48] Adams R.P. (2007). Identification of Essential Oil Components by Gas Chromatography/Mass Spectrometry.

[49] König W.A., Joulain D., Hochmuth D.H. (2004). Terpenoids and Related Constituents of Essential Oils.

[50] Jovanović D.Z., Nešić M.S., Dekić M.S., Ristić N.R., Radulović N.S. (2025). Resolving unresolvable multiplets: ^1^H NMR spectral simulation of selected sesquiterpenoids from the acorenone-rich rhizome essential oil of sweet flag, *Acorus calamus* L. J. Mol. Struct..

[51] Tsikas D. (2017). Assessment of lipid peroxidation by measuring malondialdehyde (MDA) and relatives in biological samples: Analytical and biological challenges. Anal. Biochem..

[52] Countryman P.I., Heddle J.A. (1976). The production of micronuclei from chromosome aberration in irradiated cultures of human lymphocytes. Mutat. Res..

[53] Surralles J., Xamena N., Creus A., Catalan J., Norppa H., Marcos R. (1995). Induction of micronuclei by five pyrethroid insecticides in whole-blood and isolated human lymphocyte cultures. Mutat. Res..

[54] Camelo M.R., Kehdy S.G.F., Salas E.C., Lopes T.P.M. (2008). Amifostine protection against mitomycin-induced chromosomal breakage in Fanconi anaemia lymphocytes. Molecules.

[55] (2024). Declaration of Helsinki—Ethical Principles for Medical Research Involving Human Subjects. https://www.wma.net/policies-post/wma-declaration-of-helsinki/.

[56] Law on Health Care, Official Gazette of the Republic of Serbia, No. 25/2019, 92/2023–Authentic Interpretation and 29/2025–US Decision, 40–88. https://pravno-informacioni-sistem.rs/eli/rep/sgrs/skupstina/zakon/2019/25/2/reg.

[57] (2024). Radiological Protection-Performance Criteria for Laboratories Using the Cytokinesis-Block Micronucleus (CBMN) Assay in Peripheral Blood Lymphocytes for Biological Dosimetry.

[58] Fenech M., Chang W.R., Kirsch-Volders M., Holland N., Bonassi S., Zeiger E. (2003). HUman MicronNucleus project. HUMN project: Detailed description of the scoring criteria for the cytokinesis-block micronucleus assay using isolated human lymphocyte cultures. Mutat. Res..

