# Supplementary material for: Chemical Profiles of Serbian Nepeta cataria Essential Oils, Structural Characterization of trans-cis-Nepetalactone, and Preliminary In Vitro Bioactivity
Source: Plants (Basel). 2026 Nov 2;15(21):3406. doi: 10.3390/plants15213406 (PMC13766126; doi:10.3390/plants15213406)
Supplement: Supplementary file 1 [file plants-15-03406-s001.zip › plants-4609247-supplementary.pdf]

Chemical Profiles of Serbian *Nepeta cataria* Essential Oils, Structural Characterization of *trans-cis*-Nepetalactone, and Preliminary In Vitro Bioactivity

Jelena S. Lazarević [1,*], Miroslava B. Stanković [2], Milan S. Nešić [3], Niko S. Radulović [3], Bojan K. Zlatković [4], Gordana S. Stojanović [3]

[1]Department of Chemistry, Faculty of Medicine, University of Niš, Bulevar Dr Zorana Đinđića 81, 18000 Nis, Serbia; jelena.lazarevic@medfak.ni.ac.rs; jelena217@yahoo.com
[2]Nuclear Facilities of Serbia, Mike Petrovića Alasa 12–14, 11351, Vinča, Serbia; miroslava.stankovic@nuklearniobjekti.rs
[3]Department of Chemistry, Faculty of Sciences and Mathematics, University of Niš, Višegradska 33, 18000 Niš, Serbia; milan.nesic@pmf.edu.rs (M.S.N.); nikoradulovic@yahoo.com (N.S.R.); gordanastojanovic2411960@gmail.com (G.S.S.)
[4]Department of Biology and Ecology, Faculty of Sciences and Mathematics, University of Niš, Višegradska 33, 18000 Niš, Serbia; bojan.zlatkovic@pmf.edu.rs

[*]Correspondence: jelena.lazarevic@medfak.ni.ac.rs; jelena217@yahoo.com

Supplementary material

**Figure S1.** Total ion chromatograms (TICs) of *N. cataria* essential oil samples	Pag. 2
MS scans of the iridoids (MI) identified in *N. cataria* (NC_1−NC_3) essential oils	Pag. 3-6
Data related to the isolated *trans*,*cis*-Nepetalactone; IUPAC: *rel*-(4a*S*,7*S*,7a*S*)-4,7-dimethyl-5,6,7,7a-tetrahydrocyclopenta[c]pyran-1(4a*H*)-one (4a*α*,7*α*,7a*β*-Nepetalactone; NL) from NC_1 essential oil	Pag. 7-13
**Figure S2.** Total ion chromatogram (TIC) of the isolated NL	Pag. 7
MS scan of the NL isolated from NC_1 essential oil	Pag. 7
**Table S1**. NMR spectral data of the isolated NL	Pag. 8
**Figure S3.** ^{1}H NMR (400 MHz, $CDCl_3$) spectrum of the isolated NL	Pag. 9
**Figure S4.** Comparison of the simulated and observed ^{1}H NMR (400 MHz, $CDCl_3$) spectrum of the isolated NL	Pag. 10
**Figure S5.** Selected observed ^{1}H−^{13}C coupling constants represented on the 3D model of the isolated NL	Pag. 11
**Figure S6.** ^{13}C NMR (100.6 MHz, $CDCl_3$) spectrum of the isolated NL	Pag. 12
**Figure S7.** Comparison of the simulated and observed coupled ^{13}C (100.6 MHz, $CDCl_3$) NMR spectrum of selected carbon signals of the isolated NL	Pag. 13
**Figure S8.** LP-inhibitory activity (IC_{50}, mg/L) of *N. cataria* oil samples NC_1−NC_3, isolated NL, and standards (caffeic acid and Trolox)	Pag. 14-15

NC_1

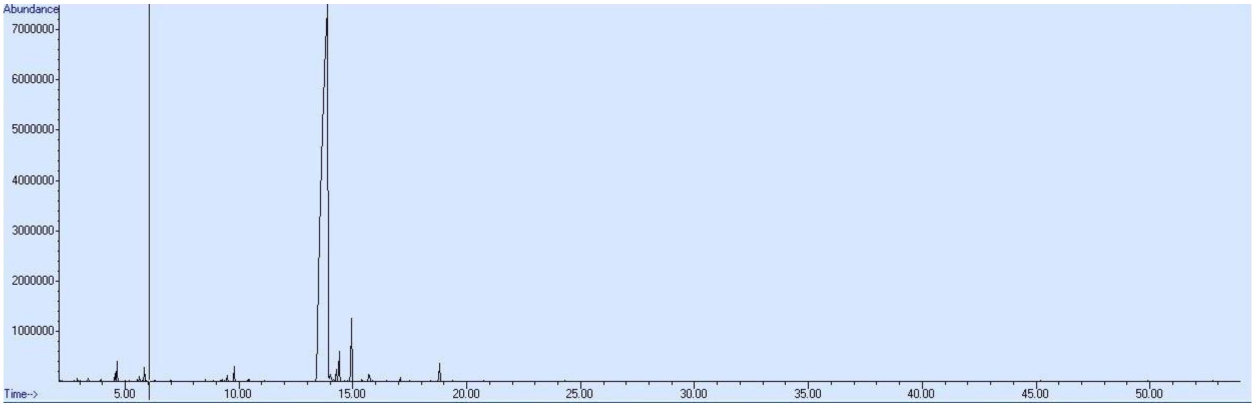

NC_2

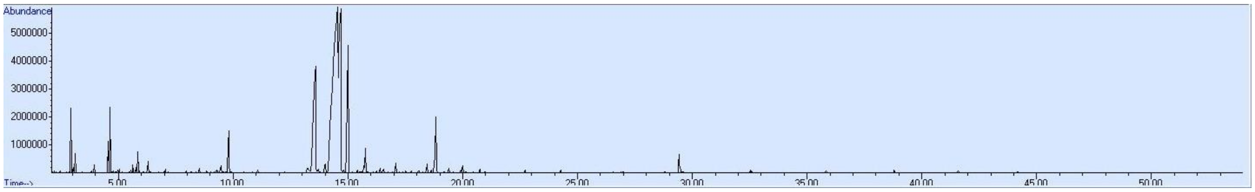

NC_3

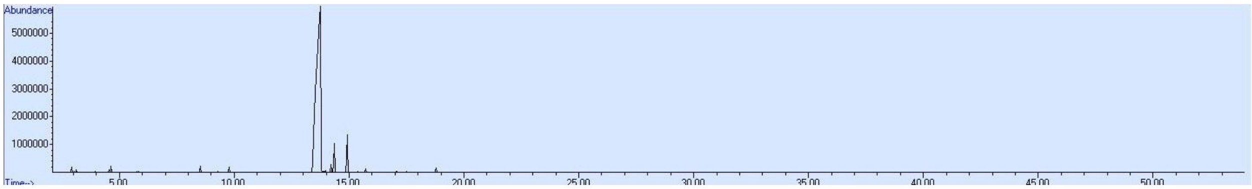

Figure S1. Total ion chromatograms (TICs) of *N. cataria* essential oil samples.

MS scans of the iridoids identified in N. cataria essential oils

Nepetalactol isomer A: RI = 1319; MS (m/z): 168 (89), 97 (94), 84 (76), 83 (77), 81 (100), 71 (82), 67 (73), 41 (97), 39 (76) [34].

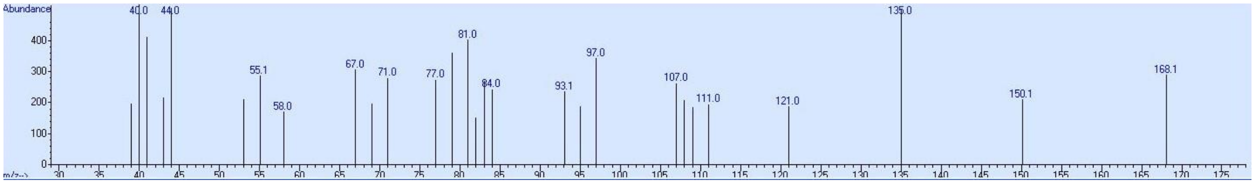

Nepetalactol isomer B: RI = 1341; MS (m/z): 168 (100), 135 (92), 108 (86), 97 (92), 79 (87), 71 (81), 67 (85), 58 (79), 55 (89), 43 (84) [34].

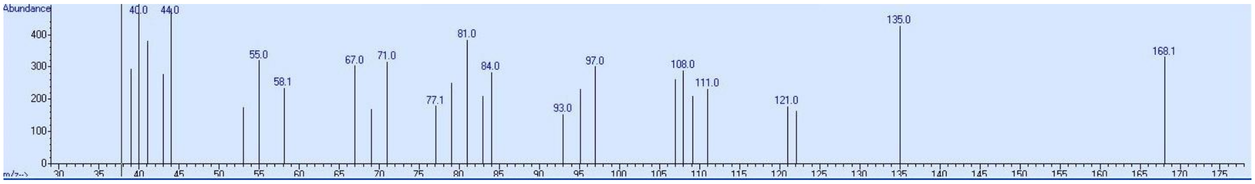

Nepetalactone isomer A: RI = 1358; MS (m/z): 166 (85), 123 (100), 109 (65), 95 (88), 81 (97), 80 (46), 69 (87), 67 (55), 41 (56), 39 (47) [35].

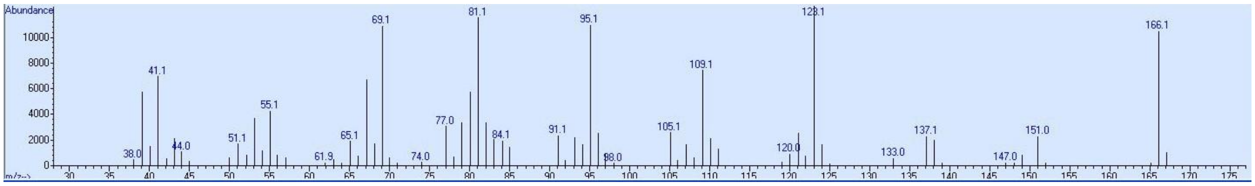

Nepetalactone isomer C: RI = 1377; MS (m/z): 166 (100), 123 (77), 109 (57), 95 (87), 81 (66), 80 (40), 69 (64), 67 (33), 41 (36), 39 (31) [35].

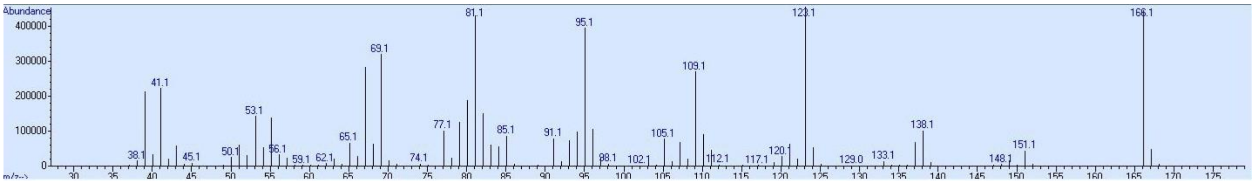

4aα,7α,7aβ-Nepetalactone, as confirmed by a high-quality EI-MS spectrum and RI match with those of the isolated NL (the achiral DB-5MS column cannot determine the specific enantiomer, NL NMR data strongly supports the relative configuration): RI = 1384; MS (m/z): 166 (98), 123 (100), 109 (62), 95 (89), 81 (99), 80 (44), 69 (75), 67 (65), 41 (49), 39 (47) [35].

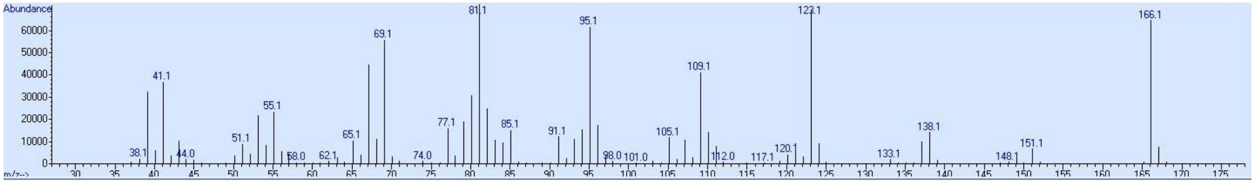

Nepetalactone isomer D: RI = 1399; MS (m/z): 166 (74), 123 (100), 109 (56), 95 (83), 81 (93), 80 (45), 69 (69), 67 (48), 41 (50), 39 (42) [35].

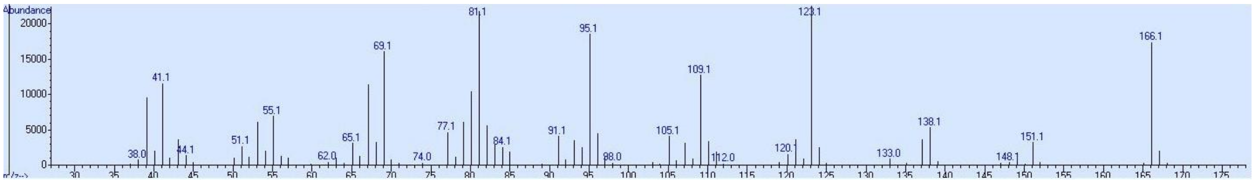

Nepetalactone isomer E: RI = 1404; MS (m/z): 166 (91), 123 (100), 109 (57), 95 (79), 81 (92), 80 (40), 69 (80), 67 (60), 41 (51), 39 (45) [35].

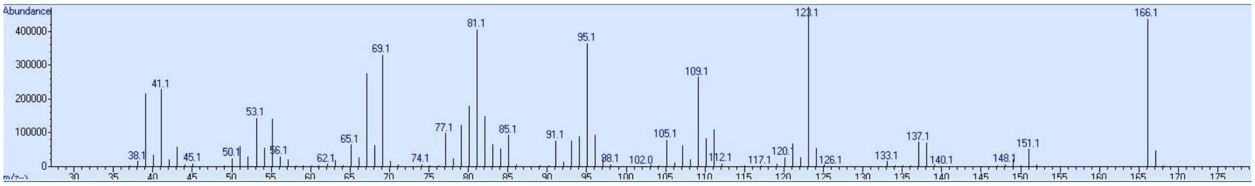

Dihydronepetalactone isomer A: RI = 1424; MS (m/z): 168 (6), 154 (6), 153 (54), 139 (4), 127 (10), 126 (18), 125 (11), 114 (12), 113 (100), 100 (6) [36-39].

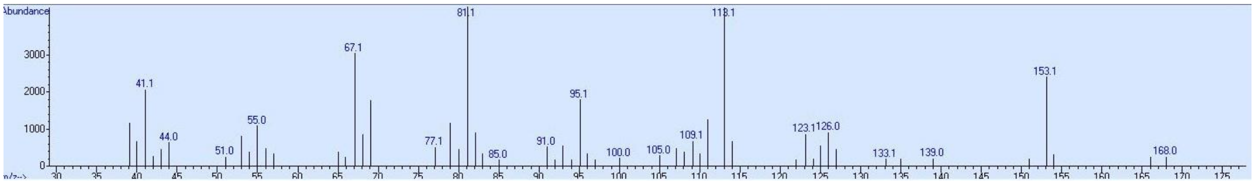

Iridomyrmecin-type compound: RI = 1445; MS (m/z): 109 (26), 95 (48), 82 (28), 81 (100), 69 (20), 68 (47), 67 (81), 55 (23), 41 (29), 39 (17) [36,37].

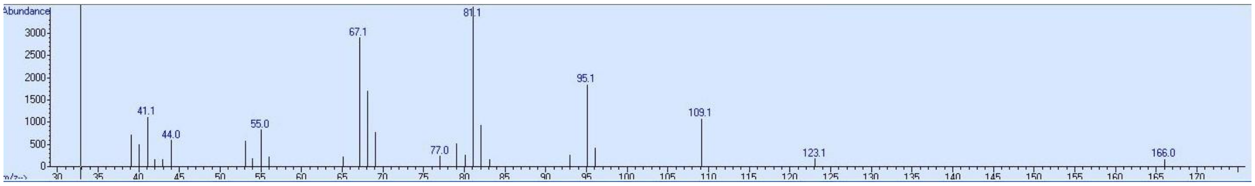

Dihydronepetalactone isomer B: RI = 1467; MS (m/z): 153 (64), 113 (58), 109 (20), 95 (30), 82 (39), 81 (100), 68 (42), 55 (37), 53 (21), 41 (35) [36-38].

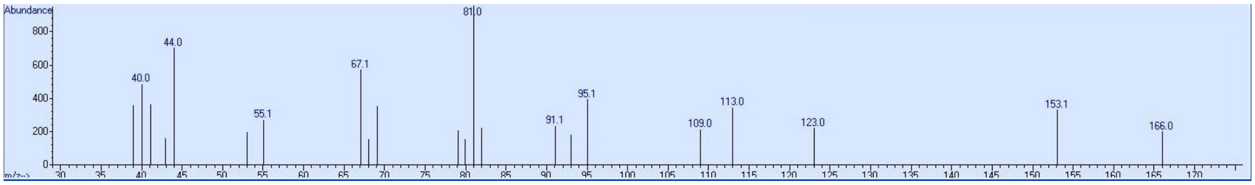

unidentified/putative iridoid

RI = 1481; MS (m/z): 109 (100), 96 (74), 87 (62), 81 (98), 67 (47), 69 (35), 55 (40), 43 (36), 41 (45), 39 (31) [37,38,40,41].

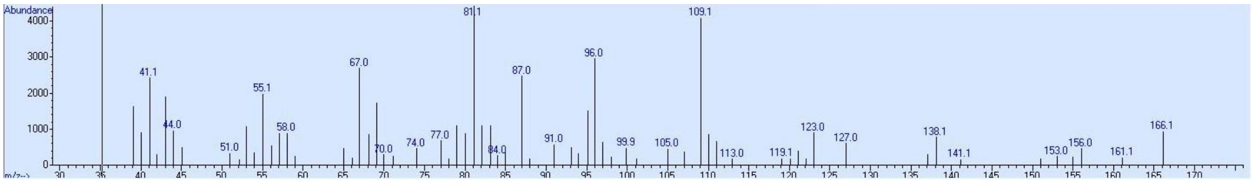

unidentified/putative iridoid

RI = 1533; MS (m/z): 109 (49), 96 (99), 87 (91), 81 (100), 82 (38), 69 (43), 67 (61), 58 (38), 55 (41), 41 (50) [37-41].

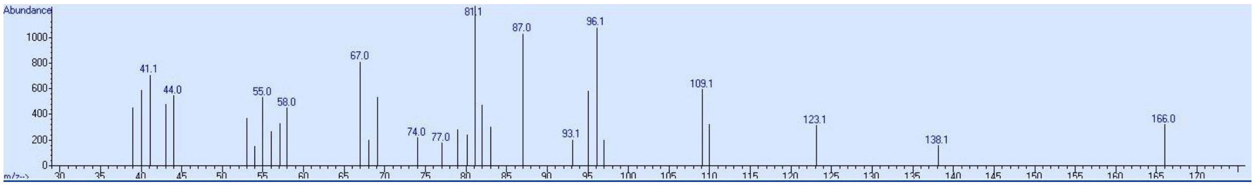

Data related to the isolated NL (*trans*,*cis*-Nepetalactone; 4a*α*,7*α*,7a*β*-Nepetalactone; IUPAC: *rel*-(4a*S*,7*S*,7a*S*)-4,7-dimethyl-5,6,7,7a-tetrahydrocyclopenta[c]pyran-1(4a*H*)-one) from NC_1 essential oil

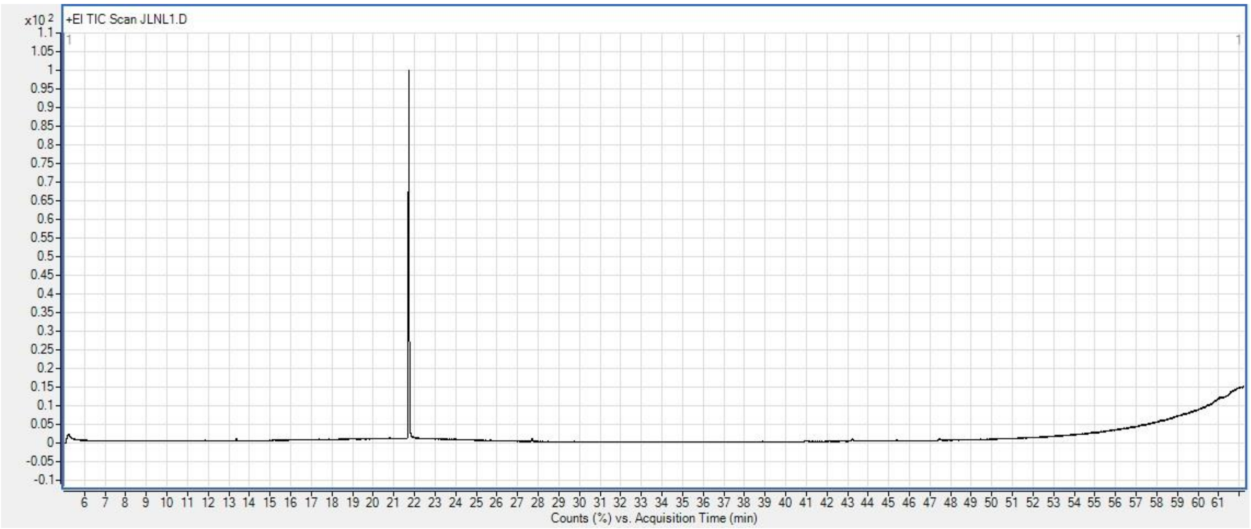

Figure S2. Total ion chromatogram (TIC) of the isolated NL

MS scan of the NL isolated from NC_1 essential oil

4a*α*,7*α*,7a*β*-Nepetalactone (NL): RI = 1384; MS (m/z): 166 (87), 123 (89), 109 (54), 95 (80), 82 (28), 81 (100), 80 (56), 69 (73), 67 (45), 55 (27) [35].

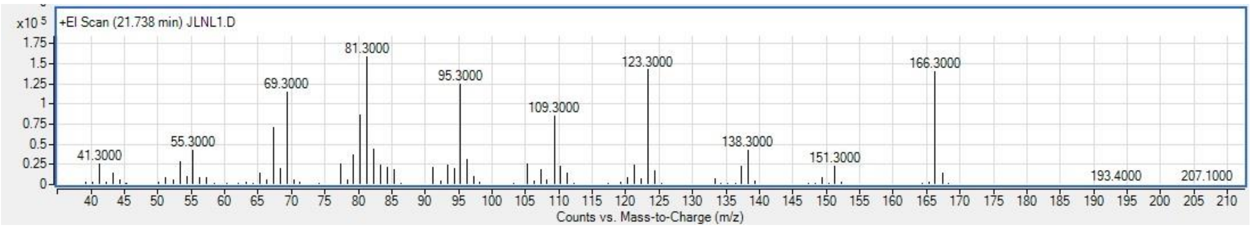

Table S1. ^{1}H (400 MHz) and ^{13}C (100.6 MHz) NMR spectral data (in $CDCl_3$) of the isolated NL (NMR parameters are derived from manual iterative full spin analysis).

Position	δ_H (m, J (Hz), Integral)[a]	δ_C (m, J (Hz))[b]	gHMBC[c]	NOESY[d]
1	/	170.33 (td, $^{2}J_{\mathrm{H-7A,C-1}}$ = 7.2, $^{3}J_{\mathrm{H-3,C-1}}$ = 4.3)	/	/
3	6.2416 (dq, $^{4}J_{3,4A}$ = −2.9, $^{4}J_{3,8}$ = −1.7, 1 H)	135.93 (dqd, $^{1}J_{\mathrm{H-3,C-3}}$ = 195.5, $^{3}J_{\mathrm{H-8,C-3}}$ = 7.6, $^{3}J_{\mathrm{H-4A,C-3}}$ = 4.3)	1, 4, 4A, 8	8
4	/	120.54 (m)	/	/
4A	2.6930 (ddddqdd, $^{3}J_{4A,7A}$ = 15.1, $^{3}J_{4A,5a}$ = 11.7, $^{3}J_{4A,5b}$ = 7.2, $^{4}J_{3,4A}$ = −2.9, $^{4}J_{4A,8}$ = −1.5, $^{4}J_{4A,6a}$ = $^{4}J_{4A,6b}$ = 0.1, 1 H)	37.39 (m[e], $^{1}J_{\mathrm{H-4A,C-4A}}$ = 125.8)	1, 3, 4, 5, 7	5b, 9
5a	1.3675 (dddd, $^{2}J_{5a,5b}$ = −12.6, $^{3}J_{4A,5a}$ = 11.7, $^{3}J_{5a,6b}$ = 10.7, $^{3}J_{5a,6a}$ = 8.5, 1 H)	26.21 (ddsext, $^{1}J_{\mathrm{H-5a,C-5}}$ = 133.4, $^{1}J_{\mathrm{H-5b,C-5}}$ = 129.1, $^{2}J_{\mathrm{H-4A,C-5}}$ = $^{2}J_{\mathrm{H-6a,C-5}}$ = $^{2}J_{\mathrm{H-6b,C-5}}$ =$^{3}J_{\mathrm{H-7,C-5}}$ = $^{3}J_{\mathrm{H-7A,C-5}}$ = 4.0)	4, 4A, 6	6b, 7A
5b	1.9640 (dddd, $^{2}J_{5a,5b}$ = −12.6, $^{3}J_{5b,6a}$ = 8.8, $^{3}J_{4A,5b}$ = 7.2, $^{3}J_{5b,6b}$ = 2.8, 1 H)		4A, 6, 7, 7A	4A, 6a
6a	1.3890 (dddddd, $^{2}J_{6a,6b}$ = -13.6, $^{3}J_{5b,6a}$ = 8.8, $^{3}J_{5a,6a}$ = 8.5, $^{3}J_{6a,7}$ = 2.7, $^{4}J_{6a,7A}$ = 0.3, $^{4}J_{4A,6a}$ = 0.1, 1 H)	32.15 (m[e], $^{1}J_{\mathrm{H-6a,C-6}}$ = $^{1}J_{\mathrm{H-6b,C-6}}$ = 130.7)	4A, 5, 7A, 9	5b
6b	2.1470 (dddddd, $^{2}J_{6a,6b}$ = −13.6, $^{3}J_{5a,6b}$ = 10.7, $^{3}J_{6b,7}$ = 8.4, $^{3}J_{5b,6b}$ = 2.8, $^{4}J_{6b,7A}$ = 0.3, $^{4}J_{4A,6b}$ = 0.1, 1 H)		4A, 5, 6, 7, 7A, 9	5a, 7
7	2.5035 (dqdd, $^{3}J_{6b,7}$ = 8.4, $^{3}J_{7,9}$ = 7.1, $^{3}J_{7,7A}$ = 6.8, $^{3}J_{6a,7}$ = 2.7, 1 H)	30.05 (m[e], $^{1}J_{\mathrm{H-7,C-7}}$ = 136.0, $^{2}J_{\mathrm{H-9,C-7}}$ = 4.7)	4A, 5, 6, 7A, 9	6b, 9
7A	2.3507 (ddddq, $^{3}J_{4A,7A}$ = 15.1, $^{3}J_{7,7A}$ = 6.8, $^{4}J_{6a,7A}$ = $^{4}J_{6b,7A}$ = $^{4}J_{7A,9}$ = 0.3, 1 H)	49.16 (m[e], $^{1}J_{\mathrm{H-7A,C-7A}}$ = 122.6)	1, 4, 4A, 5, 7, 9	5a
8	1.7153 (dd, $^{4}J_{3,8}$ = −1.7, $^{4}J_{4A,8}$ = −1.5, 3 H)	14.38 (qd, $^{1}J_{\mathrm{H-8,C-8}}$ = 127.7, $^{3}J_{\mathrm{H-3,C-8}}$ = 3.6)	3, 4, 4A,	3
9	1.1196 (dd, $^{3}J_{7,9}$ = 7.1, $^{4}J_{7A,9}$ = 0.3, 3 H)	17.70 (qdtd, $^{1}J_{\mathrm{H-9,C-9}}$ = 125.8, $^{3}J_{\mathrm{H-7A,C-9}}$ = 7.8, $^{3}J_{\mathrm{H-6a,C-9}}$ = $^{2}J_{\mathrm{H-7,C-9}}$ = 5.6, $^{3}J_{\mathrm{H-6b,C-9}}$ = 3.0)	6, 7, 7A	4A, 7

[a] Coupling constants were initially inferred from ^{1}H homoselective decoupling NMR experiments and subsequently refined by manual iterative full spin analysis. [b] ^{1}H–^{13}C coupling constants. Long-range (^{2}J and ^{3}J) couplings were tentatively assigned based on expected magnitude ranges; ^{3}J values were correlated with dihedral angles estimated from the 3D model. [c] gHMBC correlations observed between the hydrogen in this row and the carbon in the listed position. [d] Cross-peaks observed in the NOESY spectrum. [e] The main resolved coupling constants are reported, whereas the remaining couplings are unresolved.

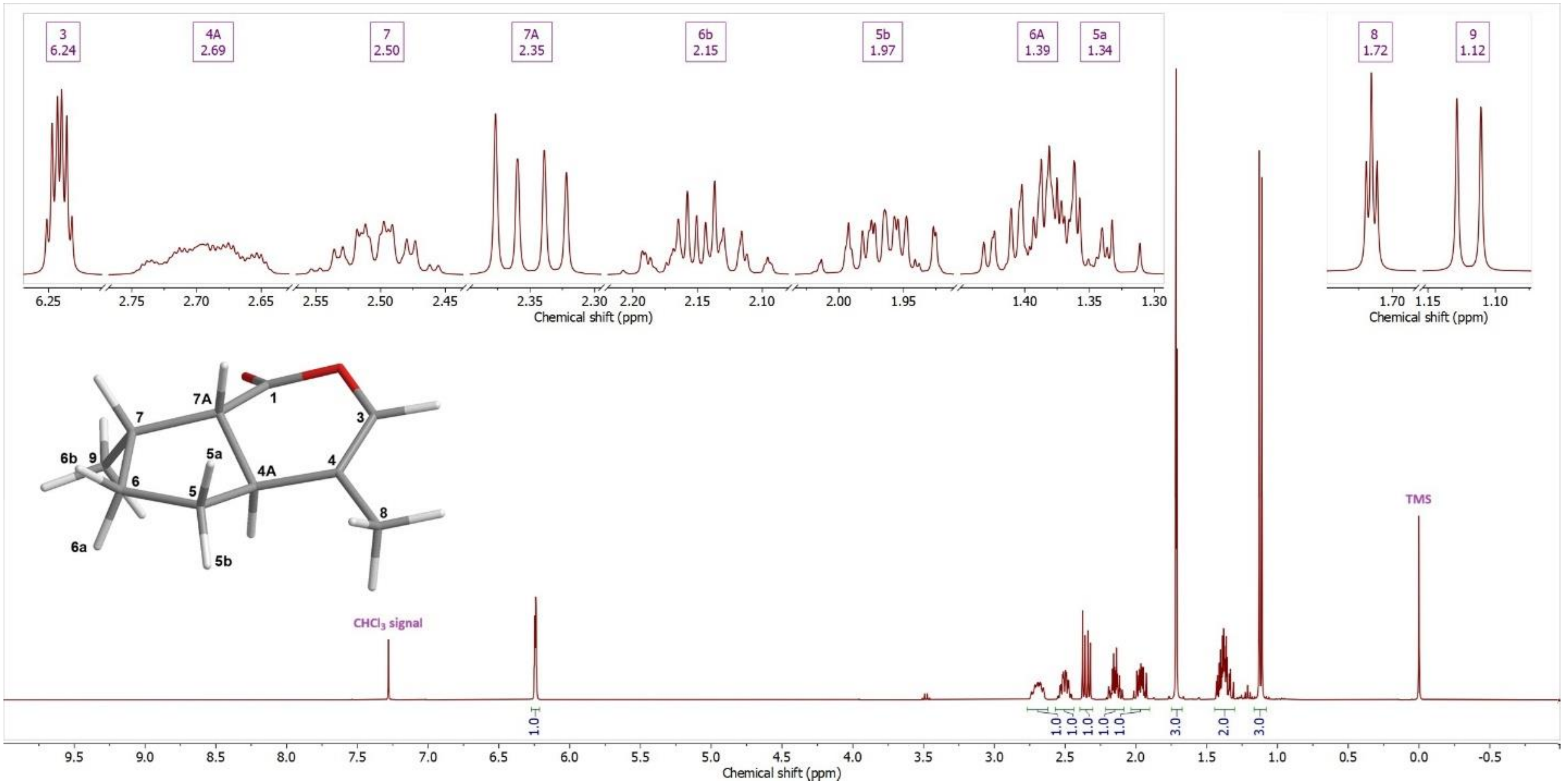

Figure S3. ^{1}H NMR (400 MHz, $CDCl_3$) spectrum of the isolated NL.

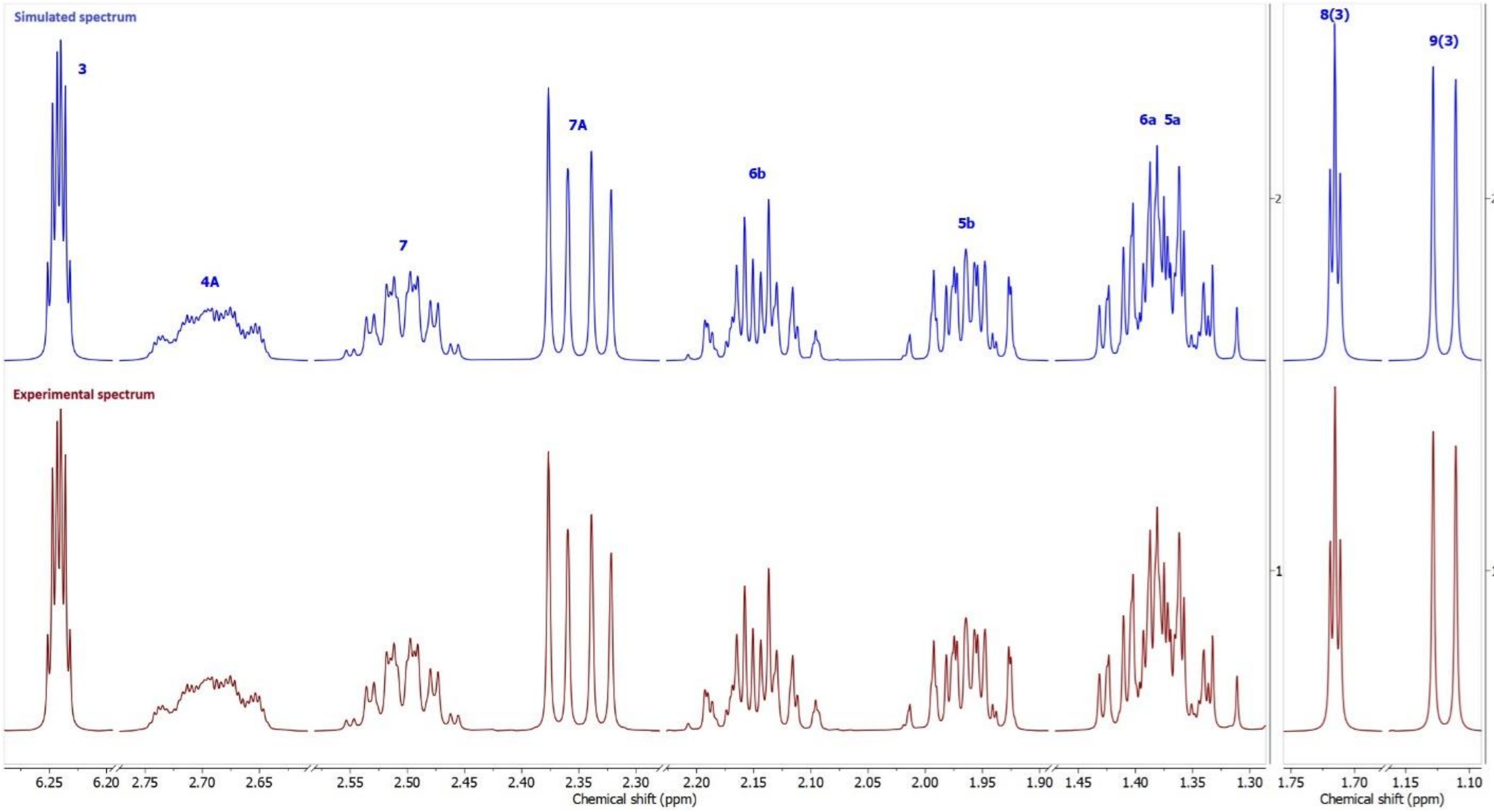

Figure S4. Comparison of the simulated and observed ^{1}H NMR (400 MHz, $CDCl_3$) spectrum of the isolated NL.

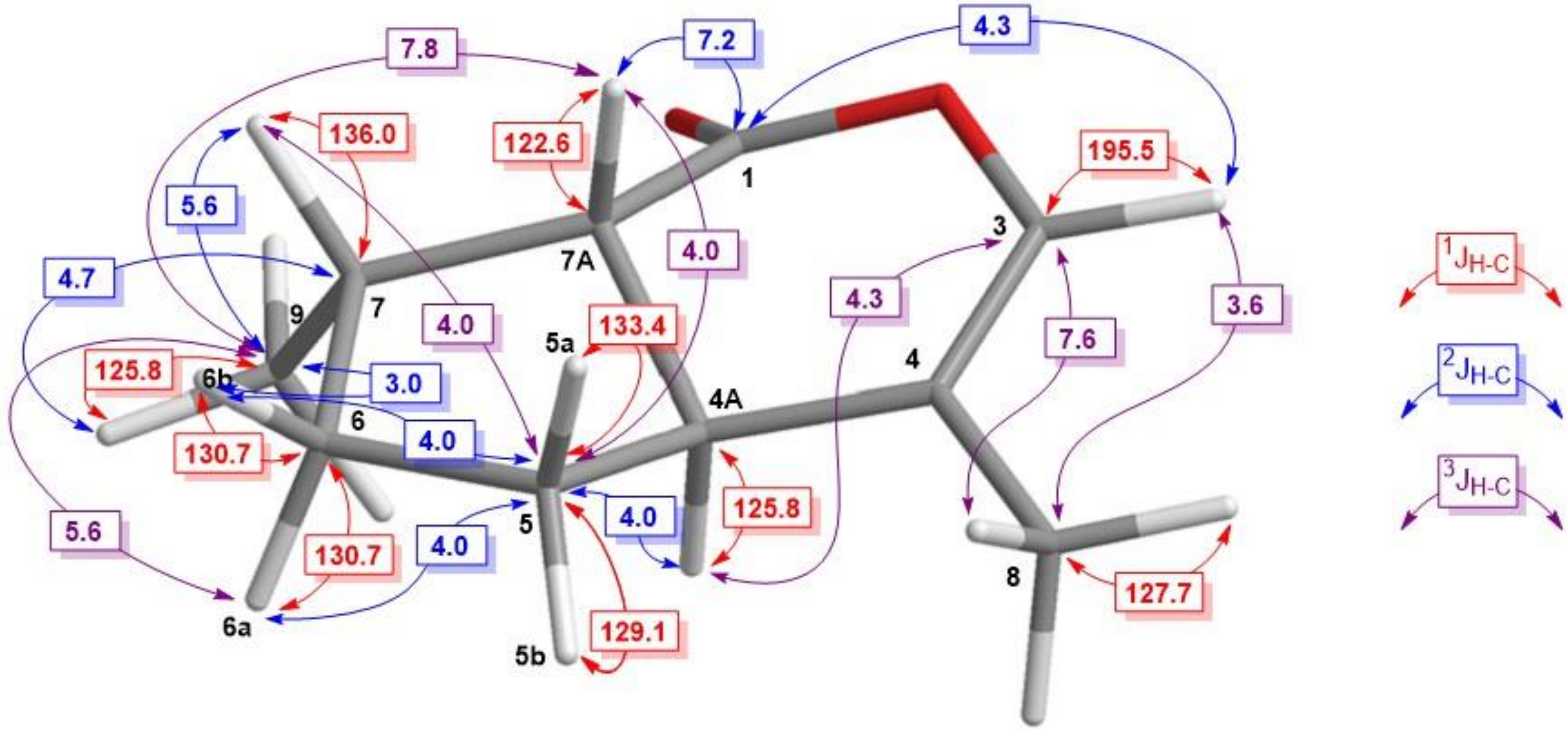

Figure S5. Selected observed ^{1}H-^{13}C coupling constants represented on the 3D model of the isolated NL.

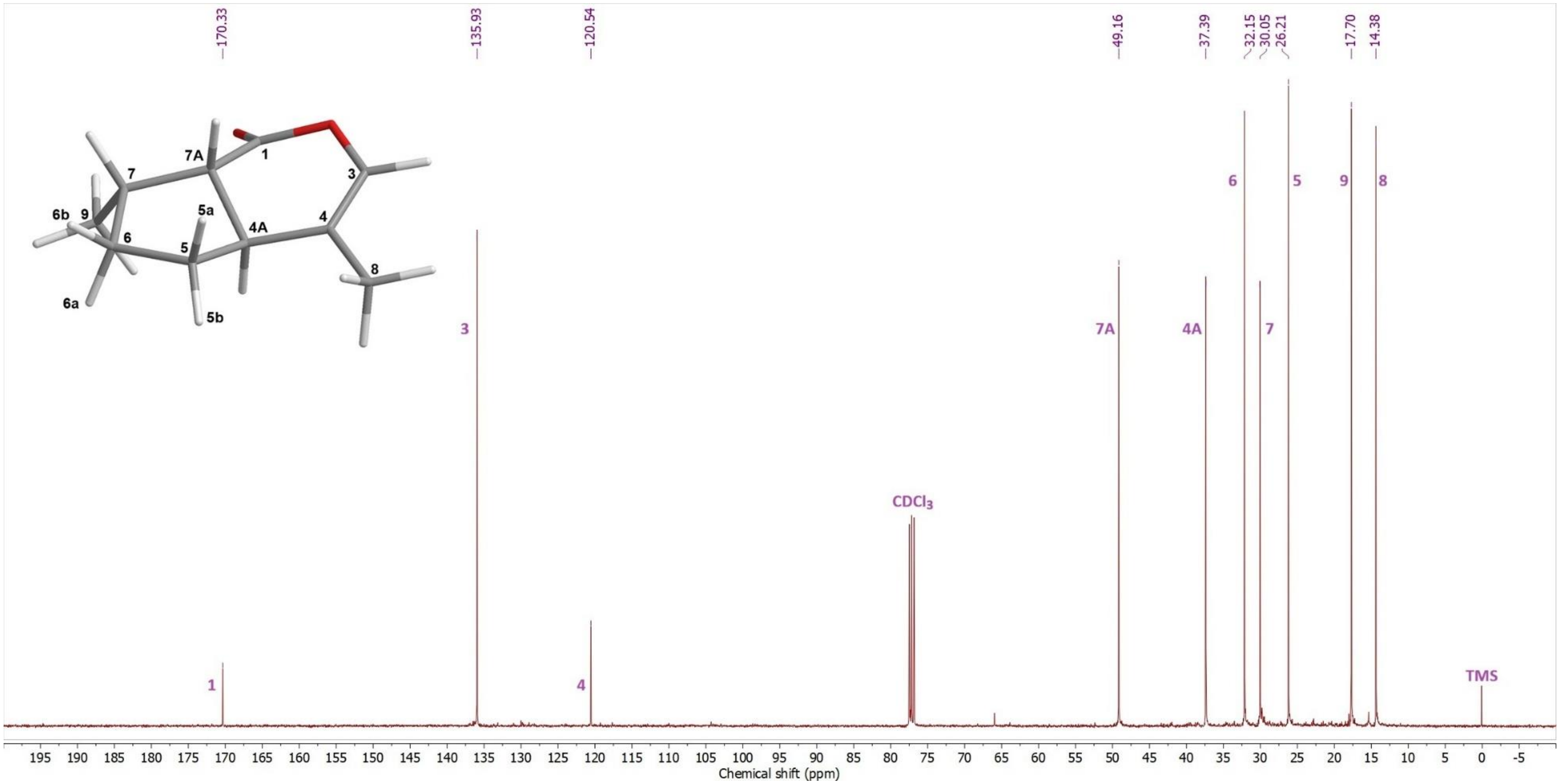

Figure S6. ^{13}C NMR (100.6 MHz, $CDCl_3$) spectrum of the isolated NL.

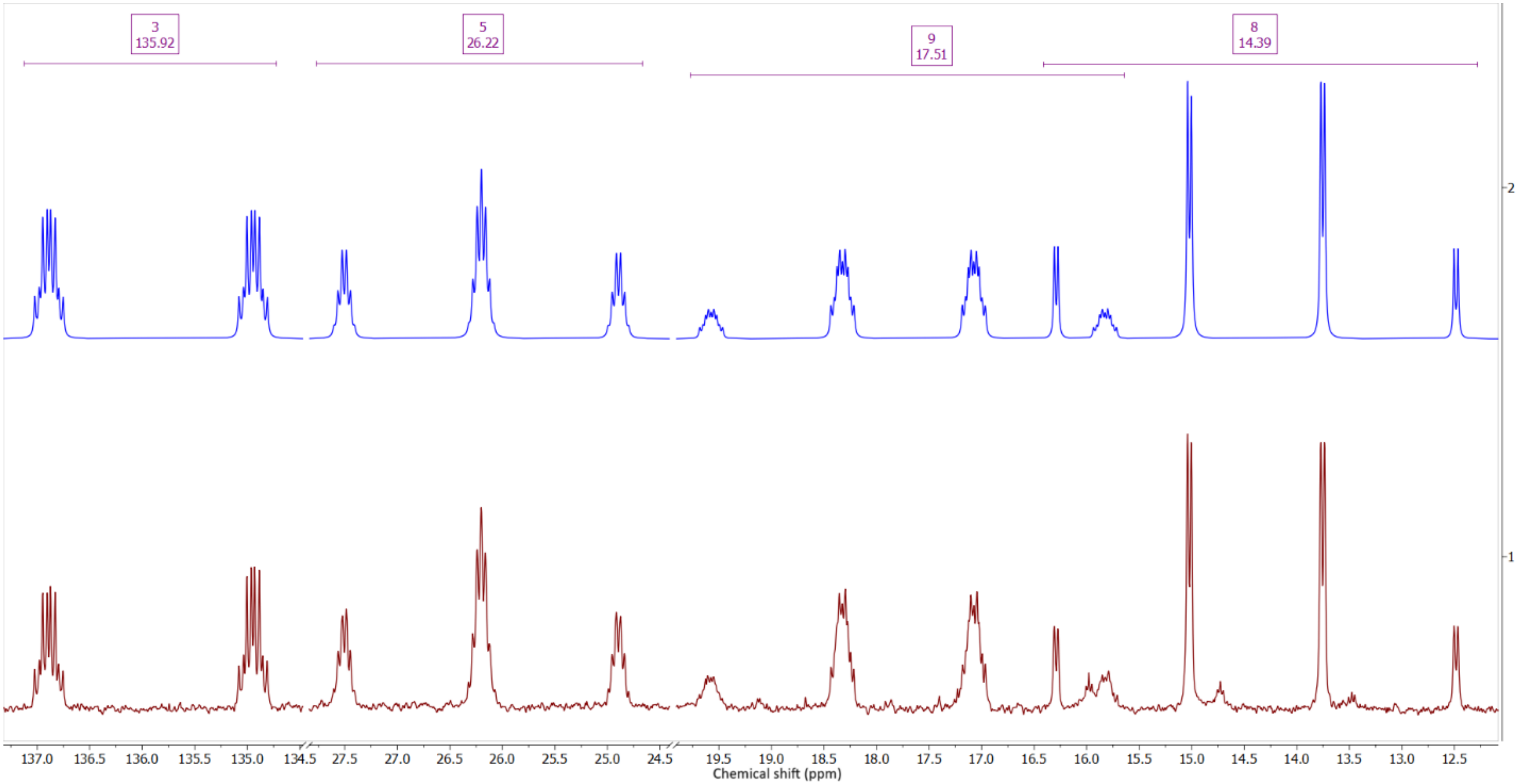

Figure S7. Comparison of the simulated and observed coupled ^{13}C (100.6 MHz, $CDCl_3$) NMR spectrum of selected carbon signals of the isolated NL.

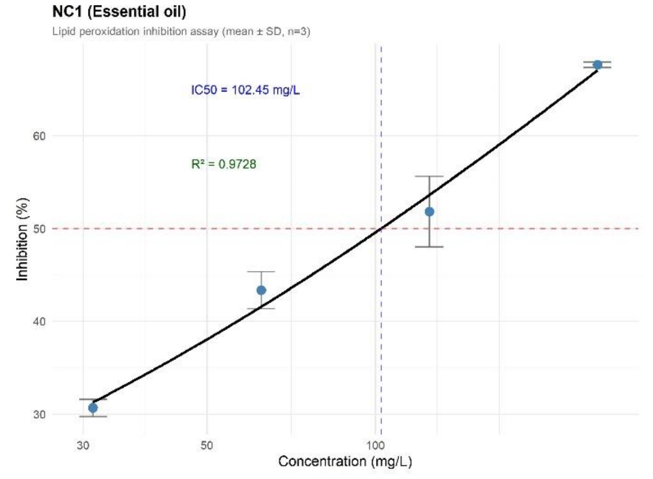

IC$_{50}$ = 102.5 mg/L, 95% CI not determined*

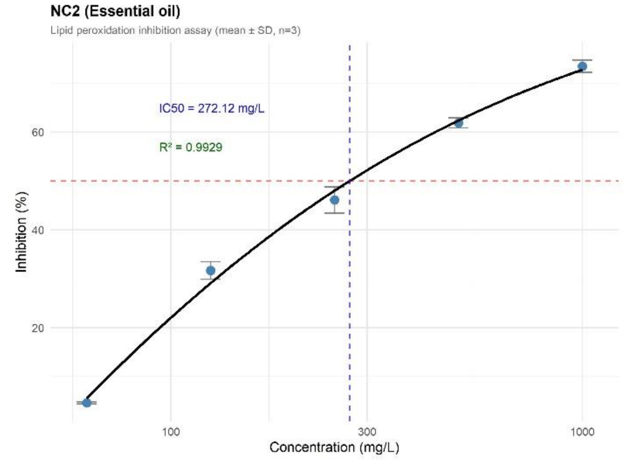

IC$_{50}$ = 272.1 mg/L, 95% CI not determined

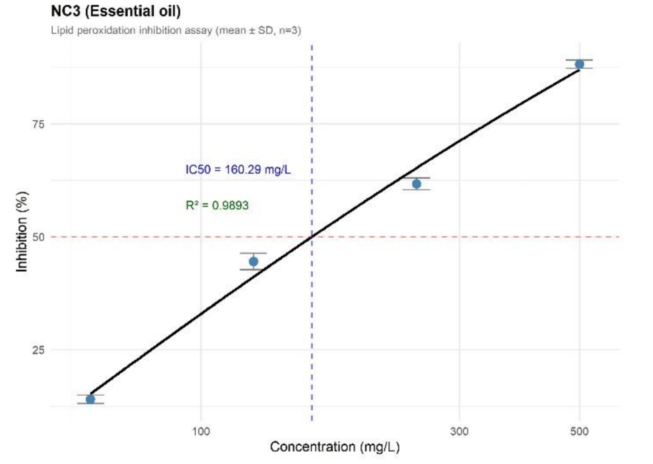

IC$_{50}$ = 160.3 mg/L, 95% CI not determined

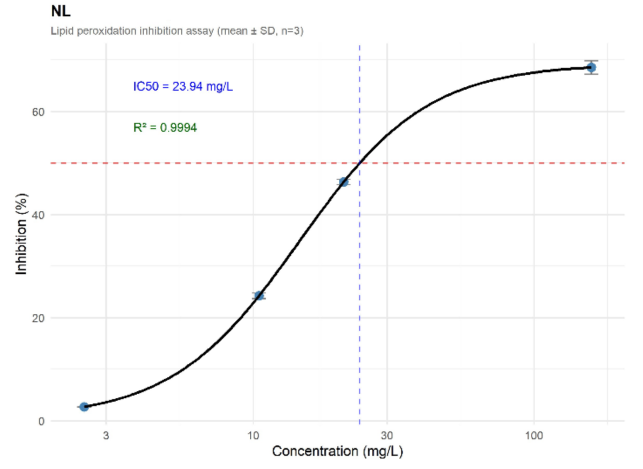

IC$_{50}$ = 23.9 mg/L, 95% CI 22.7–25.2 mg/L

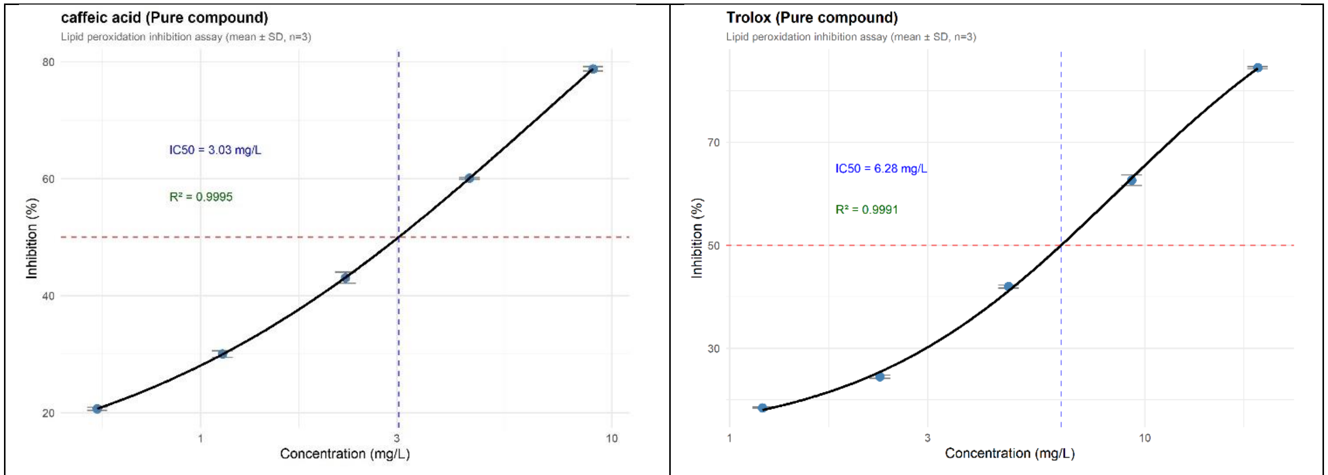

IC_{50} = 3.0 mg/L, 95% CI 2.1–4.0

IC_{50} = 6.3 mg/L, 95% CI 5.6–6.9

Figure S8. LP-inhibitory activity (IC_{50}, mg/L) of *N. cataria* oil samples NC_1–NC_3, isolated NL, and standards (caffeic acid and Trolox). For each oil sample/compound a dose-response curve is showing: experimental data points (mean ± SD) for each of the independent experiments (biological replicates, *N*=3), the fitted 4-PL curve (solid line), a horizontal line at 50% inhibition (dashed line), a vertical line indicating the IC_{50} value (dashed line) and annotaton with the IC_{50} value.*Reliable 95% CI values were obtained for NL, caffeic acid and Trolox. Conversely, for the essential oil samples (NC_1–NC_3), the 95% CIs could not be reliably determined. This limitation arose because the nonlinear regression model could not mathematically converge to generate stable intervals, owing to high standard errors, complex oil concentration-response data variability, and a limited number of data points around the IC_{50} value. All tested samples and reference compounds demonstrated a consistent fit within the 4-PL model framework ($R^2 > 0.97$).

References

34. Liu, H.; Liang, S.; Zhu, M.; Shi, W.; Xu, C.; Wei, W.; Zhan, R.; Ma, D. A fused hybrid enzyme of 8-hydroxygeraniol oxidoreductase (8HGO) from *Gardenia jasminoides* and iridoid synthase (ISY) from *Catharanthus roseus* significantly enhances nepetalactol and iridoid production. *Planta* **2024**, *259*, 62. https://doi.org/10.1007/s00425-023-04287-w

35. Hernández Lozada, N.J.; Hong, B.; Wood, J.C.; Caputi, L.; Basquin, J.; Chuang, L.; Kunert, M.; Rodríguez López, C.E.; Langley, C.; Zhao, D.; Buell, C.R.; Lichman, B.R.; O'Connor, S.E. Biocatalytic routes to stereo-divergent iridoids. *Nat. Commun.* **2022**, *13*, 4718. https://doi:10.1038/s41467-022-32414-w. Erratum in: Nat. Commun. 2022, 13, 5678. https://doi:10.1038/s41467-022-33380-z.

36. Scaffidi, A.; Algar, D.; Bohman, B.; Ghisalberti, E.; Flematti, G. Identification of the cat attractants isodihydronepetalactone and isoiridomyrmecin from *Acalypha indica*. *Aust. J. Chem.* **2016**, *69*, 169–173. https://doi:10.1071/CH15476.

37. Bol, S.; Scaffidi, A.; Bunnik, E.M.; Flematti, G.R. Behavioral differences among domestic cats in the response to cat-attracting plants and their volatile compounds reveal a potential distinct mechanism of action for actinidine. *BMC Biol.* **2022**, *20*, 192. http://doi:10.1186/s12915-022-01369-1

38. Uenoyama, R.; Miyazaki, T.; Hurst, J.L.; Beynon, R.J.; Adachi, M.; Murooka, T.; Onoda, I.; Miyazawa, Y.; Katayama, R.; Yamashita, T.; Kaneko, S.; Nishikawa, T.; Miyazaki, M. The characteristic response of domestic cats to plant iridoids allows them to gain chemical defense against mosquitoes. *Sci. Adv.* **2021**, *7*, eabd9135. https://doi:10.1126/sciadv.abd9135

39. Bol, S.; Caspers, J.; Buckingham, L.; Anderson-Shelton, G.D.; Ridgway, C.; Buffington, C.A.; Schulz, S.; Bunnik, E.M. Responsiveness of cats (Felidae) to silver vine (*Actinidia polygama*), Tatarian honeysuckle (*Lonicera tatarica*), valerian (*Valeriana officinalis*) and catnip (*Nepeta cataria*). *BMC Vet. Res.* **2017**, *13*, 70. https://doi:10.1186/s12917-017-0987-6

40. Nelson, D.L. Methylcyclopentane monoterpenes of *Nepeta cataria* and *Actinidia polygama*. Doctoral dissertation, Purdue University, West Lafayette, Indiana IN 47907, USA, 1968.

41. Sakai, T.; Nakajima, K.; Sakan, S. New Monoterpene lactones of the iridane type from *Actinidia polygama* Miq. *Bull. Chem. Soc. Jpn.* **1980**, *53*, 3683–3686. https://doi.org/10.1246/bcsj.53
